# Injectable exosome-reinforced konjac glucomannan composite hydrogel for repairing cartilage defect: activation of endogenous antioxidant pathways

**DOI:** 10.1093/rb/rbaf060

**Published:** 2025-06-17

**Authors:** Cong Ye, Jiabao Xu, Youjian Wang, Minrui Ji, Ran Tao, Fei Han, Peng Zhou

**Affiliations:** Department of Orthopaedics, Affiliated Hospital of Nantong University, Medical School of Nantong University, Nantong 226001, China; Department of Orthopaedics, Affiliated Hospital of Nantong University, Medical School of Nantong University, Nantong 226001, China; Department of Orthopaedics, Affiliated Hospital of Nantong University, Medical School of Nantong University, Nantong 226001, China; Department of Orthopaedics, Affiliated Hospital of Nantong University, Medical School of Nantong University, Nantong 226001, China; Department of Orthopaedics, Affiliated Hospital of Nantong University, Medical School of Nantong University, Nantong 226001, China; Department of Orthopaedics, Affiliated Hospital of Nantong University, Medical School of Nantong University, Nantong 226001, China; Department of Sports Medicine, The 941th Hospital of Joint Logistic Support Force of Chinese People’s Liberation Army, Xining 810000, China

**Keywords:** konjac polysaccharide, injectable hydrogel, cartilage tissue engineering

## Abstract

Enhancing the regeneration of cartilage defects remains a formidable challenge, as the dysregulated microenvironment and its crosstalk with chondrocytes play pivotal roles in impairing regeneration. In this study, we proposed a natural plant polysaccharides-derived injectable hydrogel (Exos@EKM) for adapting to irregular cartilage defects. By encapsulating stem cell-derived exosomes (Exos) into polyphenol modified methacryloylated konjac glucomannan (EKM), this hydrogel exerting a potent biological synergistic effect. First, the hydrogel demonstrates favorable biocompatibility and has the capability to modulate cellular behavior through the delivery of Exos. Additionally, it demonstrates significant chondroprotective effects and reprograms macrophages to the pro-healing state. Furthermore, konjac glucomannan and polyphenols in hydrogel synergistically activate the endogenous antioxidant capacity of chondrocytes through nuclear factor erythroid 2-related factor 2 (NRF2)-dependent pathway, thereby optimizing the biological function of Exos in regulating chondrocyte behavior and maintaining normal cartilage metabolism. In a full-thickness cartilage defect model, in vivo implantation of Exos@EKM hydrogel successfully improved cartilage regeneration and ultimately restoring knee joint functionalities. Overall, this combination of natural konjac glucomannan, polyphenols and Exos has resulted in the promotion the harmony between the microenvironment, chondrocyte and ECM. This study offers a novel approach for designing biomaterials for cartilage tissue engineering.

## Graphical abstract

**Figure:**
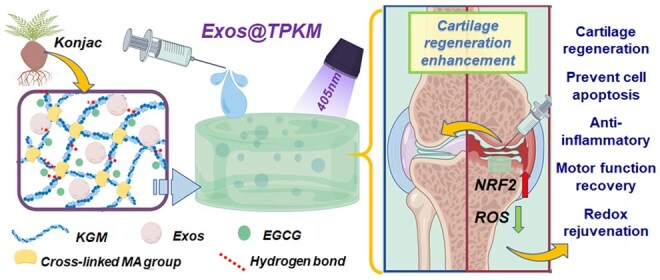

## Introduction

Articular cartilage injury is a prevalent condition, typically caused by trauma, arthritis and sports-related incidents [1, 2]. This debilitating disease is characterized by clicking of joint and subsequent loss of both surface cartilaginous tissue as well as subchondral bone integrity [3, 4]. The inflammatory response triggered by cartilage injury can initiate the endogenous repair process at the site of the defect [5]. However, this inflammatory response often leads to a high level of local oxygen free radicals (ROS), which not only directly damages the extracellular matrix but also induces cellular oxidative stress, DNA damage, and then, accelerates the degradation of cartilage matrix [5, 6]. Furthermore, overexpression of ROS exacerbates inflammation, resulting in a potent pro-inflammatory microenvironment that further impairs downstream cell function and hinders healing [7]. Timely interventions are crucial, as the absence of such measures can lead to post-traumatic osteoarthritis, thereby initiating a vicious cycle of uncontrolled inflammation and cartilage degeneration [8]. Conventional clinical approaches encompass physical therapies, nonsteroidal anti-inflammatory drugs (NSAIDs) or intra-articular injection; yet their efficacy remains constrained when attempting to bolster inherent self-repair capabilities within damaged areas [9, 10]. Despite efforts aimed at enhancing the recruitment or proliferation of chondrocytes are crucial for accelerating cartilage regeneration, it is important not to overlook how the accumulation of ROS can create hostile microenvironments that hinder proper cell differentiation and promote pathological growth patterns conducive to chronic diseases [5]. Recent research endeavors have focused on developing innovative biomaterials with specialized functionalities to reconstruct the biologically adaptive matrix for promoting cartilage tissue regeneration. For example, Shu *et al.* introduced a cobalt-doped hydroxyapatite bio-ceramic scaffold designed for scavenging ROS-induced obstacles during osteochondral repair [11]. Lu *et al*. developed a diclofenac sodium-loaded polyvinyl alcohol-based smart hydrogel which is capable of neutralizing ROS and reprogramming macrophages to reduce post-traumatic inflammation and promote cartilage regeneration [12]. In conclusion, optimizing microenvironments and promoting endogenous regenerative processes are essential for effective articular cartilage repair.

In recent years, natural polysaccharide-derived supramolecular hydrogels, such as hyaluronic acid and chitosan, have demonstrated significant potential in the field of cartilage tissue engineering [13–15]. Through covalent or noncovalent interactions, polymer molecular chains are able to self-assemble and form functional entities with notable properties of shear thinning and immediate recovery, making them ideal carriers for slow delivery and release of bioactive components to activate the endogenous healing process at sites of cartilage injury [16, 17]. Furthermore, due to the abundant functional groups often present in these substances, it is advantageous to chemically modify them to achieve various functions including injection, 3D printing, inflammation regulation and self-healing [18]. Particularly noteworthy are some naturally occurring polysaccharides that have been found to possess antioxidant and immune-modulating functions [19–21]. For instance, Zhao *et al*. reported on a fucoidan-based hydrogel that can expedite cartilage repair by activating the endogenous antioxidant pathway within cartilage tissue and stimulating the secretion of extracellular matrix (ECM) by chondrocytes [22]. Given the imbalanced oxidative microenvironment at sites of cartilage injury, natural polysaccharide hydrogels may emerge as new contenders in the realm of cartilage repair. Konjac glucomannan (KGM) is a water-soluble polysaccharide extracted from the rhizome and corm of the konjac plant. It consists of d-mannose and d-glucose units in a molar ratio approximately 1.6:1.0 linked together through beta-(1-4)-glycosidic bonds forming a framework with some branches formed by beta-(1-6)-glycosidic linkages [23]. KGM has recently been shown to exhibit anti-inflammatory and immunomodulatory properties while being considered a natural scavenger of ROS [24]. For applications in tissue engineering KGM exhibits favorable gel properties along with biodegradability; good biocompatibility; biosecurity; as well as demonstrating efficacy in skin wound healing and inflammatory diseases [25–27]. Therefore, KGM may be considered an attractive candidate for preparing scaffolds used within cartilage tissue engineering.

Although the utilization of KGM in cartilage tissue engineering or osteochondral repair is seldom reported, the exceptional antioxidant/anti-inflammatory properties of KGM are pivotal for the encapsulation of bioactive components, particularly exosomes (Exos) derived from stem cells. Exos derived from bone marrow mesenchymal stem cells have been demonstrated to stimulate cartilage tissue regeneration through pathways such as miR-23a-3p and have also exhibited certain immune modulatory effects [28, 29]. While the simulative effect of Exos on cartilage regeneration has been closely monitored, attention needs to be given to the impact of the early inflammatory stage on Exos. The harsh oxidative stress microenvironment resulting from cartilage damage may negatively affect the structure and encapsulated molecular cargo of Exos, potentially limiting their tissue regenerative effects [12, 30]. Therefore, we propose that KGM-based hydrogels could serve as an ideal carrier for Exos, effectively regulating the oxidative stress and inflammatory microenvironment to maximize their potential as a cell-free therapeutic agent for cartilage regeneration. Furthermore, for practical clinical applications, developing an injectable hydrogel would be advantageous for minimally invasive knee joint injections, not only accommodating irregular defects but also replacing open implant surgery to reduce wound size and shorten postoperative healing time. Thus, we envision that an injectable KGM-based composite hydrogel loaded with Exos can be engineered to create a niche for delivering Exos and promoting cartilage or subchondral bone regeneration by mitigating ROS and modulating immune responses.

**Figure 1.**
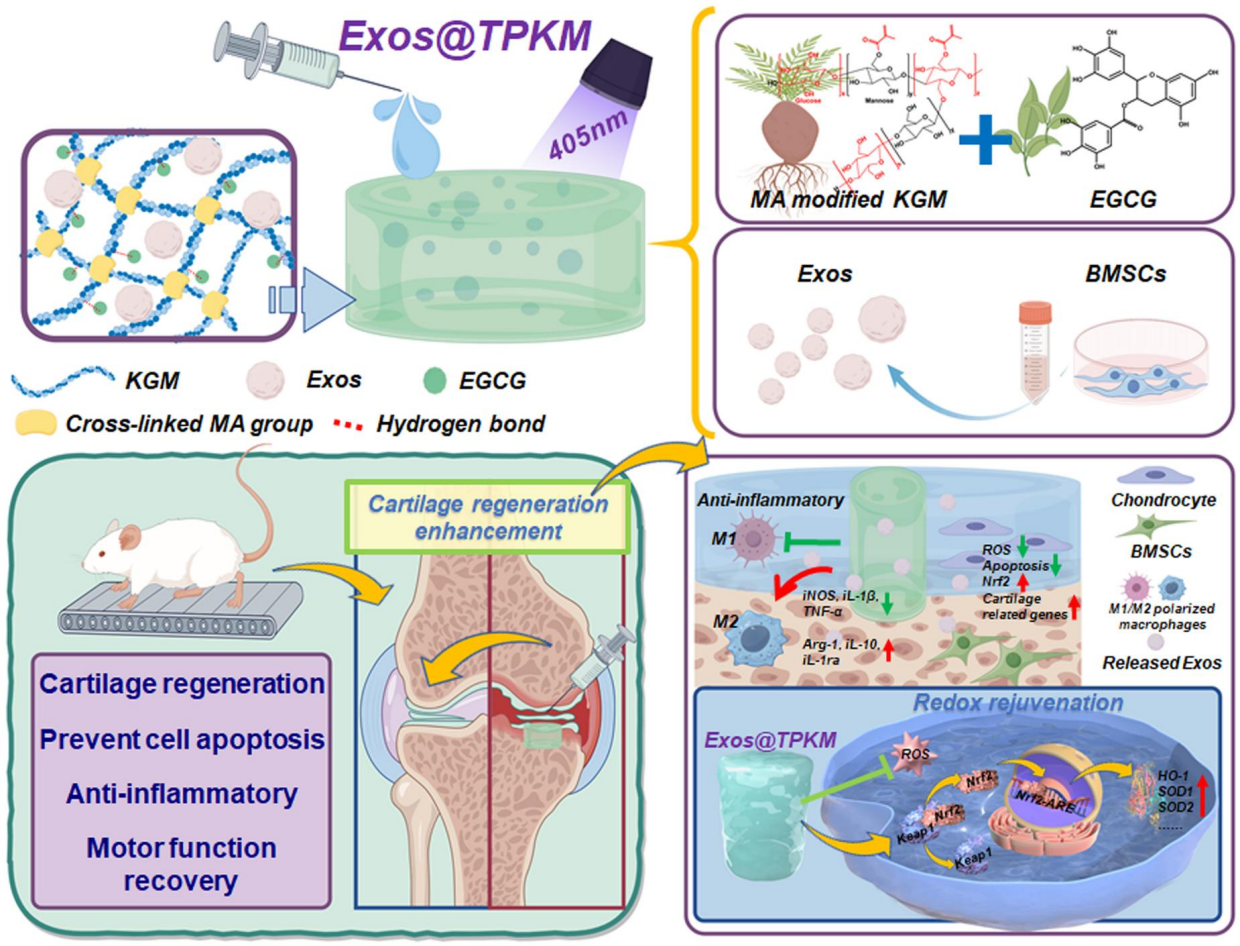
Schematic summarizing work of injectable KGM-based hydrogel (Exos@EKM), comprising KGM-, EGCG- and BMSC-derived Exos, as well as its mechanism in enhancing cartilage defect regeneration.

In this study, we have developed an Exos-loaded KGM-based injectable hydrogel (Exos@EKM) for bone cartilage repair, aiming to mitigate the adverse effects of oxidative stress microenvironment and inflammatory response on cell differentiation and proliferation, thereby enhancing the therapeutic efficiency of Exos, and addressing the limitations of methods focusing on a single stage. Specifically, KGM was modified with methacrylic anhydride (MA) to confer photo-curing properties (KGMMA), and further loaded with BMSC-derived Exos and epigallocatechin gallate (EGCG) to fabricate Exos@EKM. The interaction between polysaccharides and polyphenols allows EGCG to bind to KGMMA via hydrogen bonds, thereby enhancing the antioxidant properties of KGMMA. Following modification with MA, Exos@EKM can be cross-linked into a hydrogel by UV light irradiation, providing mechanical stability during implantation. We evaluated the role of this natural source hydrogel in improving the microenvironment and delivering Exos for promoting bone cartilage regeneration through a series of *in vitro* and *in vivo* experiments. In summary, as a natural-drived composite biomaterial, Exos@EKM has potential in improving the microenvironment and inducing tissue regeneration for promoting cartilage repair (Figure 1).

## Materials and methods

### Chemical reagents

Reagents for hydrogel synthesis, including konjac glucomannan (KGM, Mw = 500 kDa, viscosity ≥ 15 000 mPa⋅s, purity ≥ 95%), methacrylic anhydride (MA) and epigallocatechin gallate (EGCG) from Macklin (Shanghai, China), were utilized. KGM is composed of D-mannose and D-glucose, the molecular ratio of the two is 1.6:1. Reagents for cell culture, such as Minimum Essential Medium-Alpha (MEMα), fetal bovine serum (FBS) and sterile phosphate buffer solution (PBS), were obtained from Gibco (USA). All other solvents, chemical reagents and assay kits were procured commercially. The specific inhibitor of NRF2, ML385, was purchased from MedChemexpress (MCE) (USA).

### Preparation and characterization of KGMMA and EKM

The hydrogel preparation method was modified based on previous reports. Briefly, 6 g of KGM was dissolved in 120 ml of deionized water (ddH_2_O) and fully dissolved after heating in a 60°C water bath. Subsequently, 3.5 g of MA was added to the KGM solution and stirred under an ice bath for 24 h. A solution of 1 M sodium hydroxide was added dropwise to the reaction system to maintain the pH at 8.0. The resulting product underwent dialysis in ddH_2_O for 3 days and then freeze-dried to obtain KGMMA (The dialysis bag has a molecular weight cut-off of 3.5 kDa). EKM was synthesized from KGMMA. Briefly, KGMMA (4% wt) was dissolved in deionized water and different amounts of EGCG (10 μg/mL, 20 μg/ml and 30 μg/ml) were added separately. The pH was adjusted to 5.5 with a 1M acetic acid solution. After stirring for 30 min, the solution was dialyzed in deionized water in the dark for 3 days to obtain EKM. Based on the different amounts of EGCG used, the resulting products were named EKM1, EKM2 and EKM3. Additionally, Lithium Phenyl (2,4,6-trimethylbenzoyl) phosphinate (LAP) was selected as the photoinitiator for triggering UV curing of the hydrogel. The concentration of EKM hydrogel precursor solution is 4%. After the preparation of KGMMA, the methacryloyl groups on KGM were confirmed by 1H-nuclear magnetic resonance analysis (1H-NMR). Furthermore, FT-IR analysis was utilized to examine the composition of EKM. The microscopic morphology of each group of composite gels was observed using scanning electron microscope (SEM). Additionally, rheological properties and stress resistance of the hydrogels were evaluated using a rheometer. Cylindrical hydrogel samples (ø 20 mm × 4 mm) from each EKM group were prepared and affixed onto the rheometer with parameters set at 10 rad/s and shear range from 0.01% to 100%. Mechanical properties of the hydrogels were also assessed through compression tests at room temperature. Cylindrical hydrogel samples (ø 15 mm × 10 mm) were prepared, their diameter and thickness recorded, then, placed on the lower plate of a compression tester and compressed by the upper plate at a strain rate of 1 N/min. The compression modulus was calculated from the slope of the stress–strain curve obtained during compression testing (Dynamic mechanical analyzer Q800, TA, USA). The *in vitro* swelling and degradation behavior of each group of EKM hydrogels was determined based on previously reported methods [31]. For the swelling rate of the hydrogels, first, the initial weight of each group of hydrogels was measured and recorded as W_0_. Subsequently, the hydrogels were immersed in PBS at 37°C and the weight change of the hydrogels was recorded at different time points. The percentage swelling was calculated according to Equation (1) where W_t_ is the weight of the hydrogel that dissolves at a predetermined time interval.


(1) $$\mathrm{Swelling}\;\mathrm{ratio}\;\%=\frac{W_{t}-W_{0}}{W_{0}}\times 100\%.$$

The degradation ratio of the hydrogels at different time points was assessed by measuring the weight loss of samples immersed in PBS at 37°C. Over a 30-day period, the hydrogels were removed from the solution, their dry weights were measured at predetermined time intervals, and then, they were replaced with fresh PBS (containing 3 mg/ml collagenase I) for continued immersion. The percentage degradation was calculated using Equation (2). The initial dry weight (W_d0_) of each group of hydrogels was recorded, and W_dt_ represents the dry weight of the hydrogel harvested at a specific time.


(2) $$\mathrm{Degradation}\;\%=\frac{W_{d0}-W_{\mathrm{dt}}}{W_{d0}}\times 100\%.$$

The *in vitro* antioxidant properties of the hydrogels were examined by the DPPH method as previously reported. Briefly, each set of hydrogels was prepared as a cylindrical shape with a diameter of 10 mm and a thickness of 5 mm, with immersed in a configured PBS solution. Thirty  minutes later, the supernatant of the solution was taken and the UV spectra of the supernatant were analyzed using a UV spectrophotometer as a means of analyzing the free radical scavenging efficiency.

### Cell culture and cytocompatibility analysis

Rat chondrocytes were isolated from rat knee hyaline cartilage using a previously established method. Rat bone marrow stem cells (BMSCs) and macrophages (RAW 264.7) were obtained from OriCell (Guangzhou, China), and the culture medium was MEMα supplemented with 10% FBS and 1% penicillin-streptomycin (P/S, Gibco, USA). The impact of each EKM group on cell proliferation was assessed using the CCK-8 assay. Specifically, 500 μl of each gel precursor group was evenly distributed onto the bottom of a 24-well plate and cross-linked by UV irradiation, followed by seeding approximately 5 × 10^4^ chondrocytes per well. The cells were cultured on the gels for 1, 3 and 7 days, and the absorbance of each well's working solution at 450 nm was measured using a CCK-8 kit (Dojindo, Japan), following the manufacturer's instructions (*n* = 4).

### Extraction and internalization of Exos

Exosomes (Exos) were isolated from BMSCs obtained from Oricell (Guangzhou, China). Briefly, Exos were extracted from the supernatant of BMSCs culture medium using a classical gradient centrifugation method. The Extracellular Vesicle Extraction and Purification Kit (Umibio, Shanghai, China) was utilized to facilitate the isolation and purification of Exos according to the manufacturer's instructions, with a centrifugation speed ranging from 1000 to 200 000 rad/s. The protein concentration of Exos was quantified using a BCA protein concentration assay kit (Beyotime, China). Subsequently, EKM hydrogels were loaded with varying concentrations of Exos (0 µg/ml, 50 µg/ml, 150 µg/ml, 250 µg/ml, 350 µg/ml, 500 µg/ml) for culturing chondrocytes, which were designated as 1-Exos@EKM, 2-Exos@EKM, 3-Exos@EKM, 4-Exos@EKM, 5-Exos@EKM and 6-Exos@EKM, respectively. The influence of different exosome concentrations on chondrocyte matrix secretion was assessed using Alcian Blue staining, while the viability and glycosaminoglycan (GAGs) content of chondrocytes were evaluated by measuring absorbance at 630 nm. Based on this, the optimal concentration of Exos-loaded hydrogel was determined and subsequently utilized in follow-up experiments.

The Exos were subsequently characterized. First, the morphology of Exos was observed using transmission electron microscopy (TEM), and the signature proteins HSP70, TSG101 and CD9 of Exos were validated using Western Blot method (antibodies purchased from Proteintech, Wuhan, China). UCMSCs culture medium was used as the control group. Then, Exos were mixed with the precursor solution of EKM in a certain proportion and after UV curing, Exos@EKM was obtained. For internalization studies, harvested Exos were first fluorescently labeled using an ExoSparkler Exosome Membrane Labeling Kit-Red (Dojindo, Japan), followed by construction of fluorescently labeled Exos containing Exos@EKM Hydrogel. Based on a Transwell system, Exos@EKM samples (ø10 mm × 5 mm) were placed in the upper chamber and rat bone marrow mesenchymal stem cells (BMSCs) or rat chondrocytes were cultured in the lower chamber. After co-culturing for 3 days, red fluorescence inside the cells was observed using a fluorescence microscope to verify internalization of extracellular vesicles within the cells. The cytoskeleton and nucleus were labeled using phalloidine staining solution (Thermo Fisher, USA) and DAPI assay kit (Dojindo, Japan), respectively. CCK-8 and Live-Dead Stain Kit (Dojindo, Japan) were used to verify the effect of hydrogels on cell viability, and the cell culture procedure was the same as above.

### Chondroprotective and subchondral bone protective effects of Exos@EKM hydrogel

First, the capacity of hydrogels to eliminate excessive ROS in cells was assessed. Briefly, cells were seeded onto the gel as per the aforementioned method and then exposed to a culture medium containing hydrogen peroxide (200 μM) for 12 h. Subsequently, after cell digestion and collection, the level of ROS within the cells was determined using the DCFH-DA fluorescent probe kit (Dojindo, Japan) through immunofluorescence and flow cytometry analysis. Furthermore, cell apoptosis levels were assessed using a cell apoptosis detection kit (Dojindo, Japan). The activation of NRF2-related signaling pathway in the cells was evaluated via Western Blot analysis. In simple terms, nuclear and total proteins extracted separately from the collected cells were subjected to Western Blot analysis to determine expression levels of nuclear NRF2, SOD1, HO-1 and SOD2 proteins. Antibodies utilized in this study were procured from Proteintech (Wuhan, China), with detailed information listed in Supplementary Table S1. The impact of the hydrogel on chondrocyte phenotype and matrix secretion was examined by qPCR analysis. Expression levels of Sox 9, COL-2 and ACAN genes in chondrocytes were analyzed using qPCR after culturing on the gel for 7 days. The primer sequences utilized for the qPCR analysis are detailed in Supplementary Table S2. Reverse transcription kit (Vazyme Nanjing China) along with RT-PCR kit (Vazyme Nanjing China) were employed for RNA extraction followed by real-time PCR utilizing QuantStudio Q7 system (Appliedbiosystems USA). Results will be evaluated using Ct (2^^-ΔΔCt^) method to assess gene expression relative to housekeeping gene GAPDH. Additionally, Transwell application was used for identification of cartilage differentiation marker Aggrecan (ACAN) expression through cell immunofluorescence staining (IF). Moreover, Alcian blue staining (Solabio, Beijing, China) was performed on analyzing gel's effect on cartilage matrix secretion.

This study verified the subchondral bone protective effect of hydrogel *in vitro*. Briefly, BMSCs were seeded onto the hydrogel as per the aforementioned method and then exposed to a culture medium containing hydrogen peroxide (200 μM) for 12 h. Afterwards, the culture medium was replaced with osteogenic differentiation induction medium (OriCell, Guangzhou, China). Subsequently, the impact of the hydrogel on osteogenic differentiation was examined by qPCR analysis. Expression levels of COL-1, Runx2 and OPN genes in BMSCs were analyzed using qPCR after culturing on the gel for 7 days. The experimental protocol for qPCR aligns with the methodology described in the preceding section. Results will be evaluated using Ct (2^^-ΔΔCt^) method to assess gene expression relative to housekeeping gene GAPDH. Additionally, Transwell application was used for identification of osteogenic differentiation marker OPN expression through cell immunofluorescence staining (IF). Moreover, alizarin red staining kit (Solabio, Beijing, China) and alkaline phosphatase (Beyotime, Shanghai, China) were performed on analyzing hydrogel's effect on osteogenic differentiation.

### Function of hydrogel in regulating immune reprogramming

The RAW 264.7 cell line (OriCell) was employed as an experimental model to investigate the regulatory effects of Exos@EKM on macrophage polarization. In this study, a 24-well Transwell system was utilized, wherein 2 × 10^5^ RAW 264.7 cells were seeded in the lower chamber. To simulate the inflammatory microenvironment associated with cartilage injury, lipopolysaccharide (LPS, 1 μg/ml, Solarbio, Beijing, China) was added to the culture medium to stimulate the cells for 6 h, thereby inducing M1 polarization of macrophages. Subsequently, Exos@EKM samples (ø10 mm × 5 mm) were placed in the upper chamber of the Transwell system and co-cultured with the cells for 7 days under standard culture conditions. The expression levels of pro-inflammatory and M1 polarization-related genes (iNOS, IL-1β, TNF-α) as well as anti-inflammatory and M2 polarization-related genes (Arg-1, IL-10, IL-1ra) were analyzed using qPCR. Additionally, immunofluorescence labeling was performed to detect iNOS and Arg-1 proteins in RAW 264.7 cells.

### *In vivo* experiments

All animal experiments were ethically and scientifically approved by the Animal Care and Use Committee of Nantong University (Approval No.: IACUC20240220-006). All experimental procedures complied with the ARRIVE guidelines and adhered to the UK Animals (Scientific Procedures) Act 1986 and associated guidelines. Full-thickness cartilage defect models were prepared using 12-week-old female SD rats weighing 300–400 g. The rats were anesthetized with isoflurane gas and sterilized. Bilateral joint capsules were incised from the medial edge of the patella to expose the articular cartilage on the patellofemoral joint surface. Subsequently, full-thickness cartilage defects measuring 1.5 mm in diameter and 1 mm in depth were created 2 mm above the intercondylar fossa. After modeling, the joint cavity was rinsed, and both joint capsule and skin incisions were sutured. A total of 20 rats were used, and they were allowed unrestricted movement within their cages. The animals were randomly assigned to four groups: Blank (untreated), KGMMA, EKM and Exos@EKM, with 5 rats per group (*n* = 5). Precursor solutions for KGMMA, EKM and Exos@EKM were prepared, injected into the defects and crosslinked under ultraviolet light. The untreated animal models served as the Blank group. Two treatment time points were evaluated: 4 weeks and 8 weeks (*n* = 5 for each time point). At 4 and 8 weeks postoperatively, the rats were euthanized via intraperitoneal injection of pentobarbital sodium (150 mg/kg), and the distal femoral condyles of the knee joints were collected for analysis of cartilage defect repair.

### Histological and immunohistochemical analysis

Knee samples from all animal models were collected at 4 and 8 weeks postsurgery, fixed in neutral formalin for 72 h and subsequently decalcified in ethylenediaminetetraacetic acid (EDTA) solution at room temperature for one month. The samples were then dehydrated, embedded in paraffin and sectioned along the coronal plane. Regeneration of the osteochondral defects was evaluated using toluidine blue staining, safranin O/fast green staining and HE staining. Three independent images were blindly scored by experienced researchers based on the pathological data according to a modified Mankin scoring system [32]. The levels of cartilage matrix (Collagen II, COL-2) and scar matrix (Collagen I, COL-1) in the specimens were verified using immunohistochemical analysis, as well as the *in vivo* expression of NRF2. Immunofluorescence staining was used to analyze the levels of inflammation-related proteins (iNOS) and anti-inflammation-related proteins (Arg-1) in the samples after paraffin embedding and sectioning. All immunohistochemical and immunofluorescence images were quantitatively analyzed using ImageJ software. The region of interest was set as the cartilage defect area in the model, with an area size of 500 μm × 300 μm. Quantitative analysis was performed based on three independent images.

### Statistical analysis

All the quantitative data were analysed via SPSS version 18.0 (Chicago, IL, USA) and are expressed as the means ± standard deviations (SDs). Statistical significance among three or more groups was determined using a one-way analysis of variance (ANOVA) followed by the Tukey's *post hoc* test. Statistical comparisons between two groups were performed using Student's t-test. Statistical significance was indicated as ∗∗∗*P* < 0.001, ∗∗ *P* < 0.01 and ∗ *P* < 0.05.

## Results and discussion

### Preparation and characterization of konjac-derived injectable EKM hydrogel

**Figure 2.**
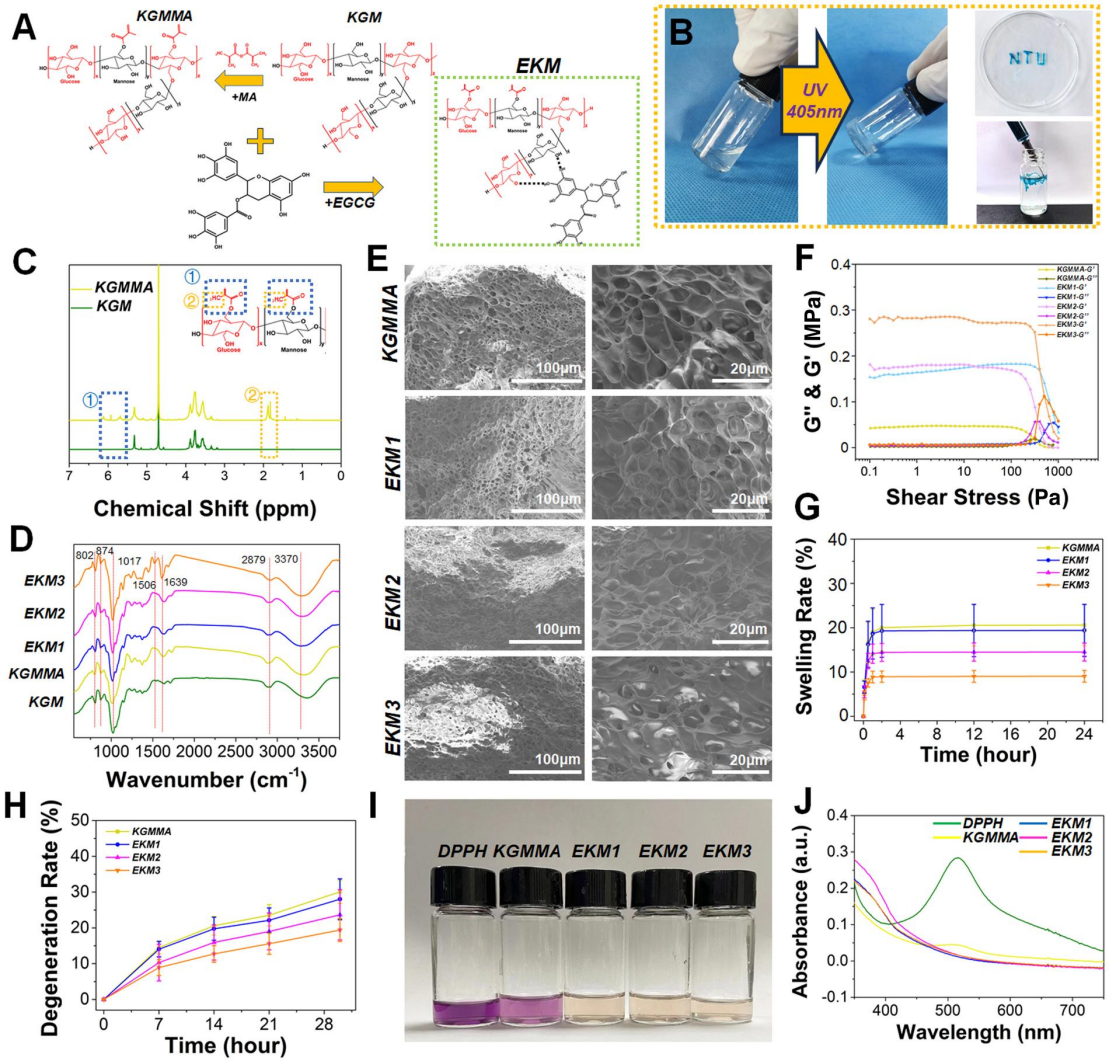
Preparation and characterization of EKM hydrogels were conducted, with EKM being cross-linked under UV light and demonstrating injectable properties. (**A**) Schematic image of the synthesis of methacrylated konjac glucomannan (KGMMA) and EGCG modified KGMMA, the EGCG molecule forms a hydrogen bond with KGMMA, resulting in the synthesis of EKM. (**B**) EKM demonstrates good injectability and gelling ability. (**C**) ^1^H NMR spectroscopy also confirms the successful synthesis of KGMMA. The blue dashed box represents the characteristic peak of the methacryloyl group, and the orange dashed box represents that of the methyl group. (**D**)–(**J**) Subsequently, the hydrogels from each group of EKM were thoroughly characterized through (**D**) FTIR spectra analysis, (**E**) SEM imaging, (**F**) rheological property testing, (**G**) swelling rate assessment in a liquid environment, (**H)** degradation characteristic evaluation, as well as (**I**) and (**J**) photos/UV–Vis spectra examination for DPPH· radical scavenging activity.

The chemical formula depicted in Figure 2A illustrates the methacryloylation of hydroxyl groups on the KGM polysaccharide units, enabling molecular cross-linking of KGM through photoinitiators and UV light (Supplementary Figure S1). Subsequently, EGCG-modified KGMMA (EKM) was synthesized, wherein EGCG and KGM interacted to form hydrogen bonds between their respective hydroxyl groups, resulting in the loading of EGCG onto the molecular chain of KGM (Figure 2A). As depicted in Figure 2B, the KGMMA synthesized in this investigation demonstrated exceptional formability and injectability even in a humid environment. This suggests that KGMMA, serving as a convenient carrier material, holds potential for filling irregular cartilage defects. The 1H-NMR spectrum illustrated in Figure 2C also confirms the successful synthesis of KGMMA, with absorption peaks at 5.7 and 6.1 ppm corresponding to the methacryloyl groups [33]. In addition, the 1H-NMR spectrum of KGMMA exhibits a distinct absorption peak at 1.9 ppm, which can be ascribed to the methyl group (-CH_3_) in the methacryloyl (MA) moiety introduced during the modification process. During the methacryloylation reaction, new methyl groups are incorporated into the molecular structure, typically manifesting as emerging absorption peak within the range of 1.8–2.0 ppm [34, 35]. The appearance or enhancement of the peak at 1.9 ppm, thus, serves as a definitive indicator of the successful introduction of methacryloyl groups onto the KGM molecular chain, thereby confirming the synthesis of KGMMA. As shown in Figure 2D, the successful synthesis of EKM was confirmed by FT-IR spectrum analysis, which revealed gradually increasing characteristic peaks at 1506 cm^−1^ and 1639 cm^−1^ in the EKM1 to EKM3 groups representing the aromatic ring structure of EGCG [36]. Additionally, in all groups, the characteristics at wavenumbers around 802 cm^−1^ and 874 cm^−1^ represent the bands of mannose in KGMMA [37], while the characteristics peaks at the wavenumbers of 1017 cm^−1^, 2879 cm^−1^ and 3370 cm^−1^ were related to C-O-C stretching vibration, C-H stretching vibration and the O-H stretching vibration, respectively [37]. Scanning electron microscopy (SEM) images illustrated uniform porous microstructures in all hydrogels (Figure 2E), which are conducive to scaffold-based cell migration and tissue growth. Meanwhile, the EKM-hydrogel exhibited solid like viscoelasticity with higher storage modulus (G′) and lower loss modulus (G′′) as shown in the rheological experiments (Figure 2F), indicating the successful gelation of EKM after UV irradiation. Notably, an increase in EGCG content led to enhanced elasticity and crosslinking density within the hydrogels. The mechanical modulus of EKM was significantly enhanced after the addition of EGCG, as evidenced by the compression test (Supplementary Figure S2), attributed to the formation of hydrogen bonding between EGCG and KGM [38], which further stabilized the hydrogel. This was also supported by the creep rate data, with all EKM samples reaching a stable state within 2 h in a liquid environment and exhibiting lower swelling rates (Figure 2G). The mechanical stability of EKM can provide structural support for damaged cartilage tissue, effectively transmit mechanical forces, and maintain shape and integrity during joint cartilage healing, while minimizing hydrogel swelling-induced compression on surrounding tissues or separation from the target organ.

Subsequently, the degradation rate of KGMMA reached 30% after 30 days of exposure to PBS, while the degradation rate of EKM decreased with increasing EGCG content in the hydrogel, reaching 20.1% for EKM2 and 16.5% for EKM3 (Figure 2H). We determined the *in vitro* free radical scavenging efficiency of each gel using the DPPH· assay. As shown in Figure 2I, the purple liquid in the KGMMA group faded, indicating the antioxidant ability of KGMMA itself, while the purple liquid in the EKM group completely disappeared. As shown in Figure 2J, the UV spectrum data showed that a weak absorption peak was still visible at 520 nm in the KGMMA group, indicating incomplete DPPH· removal. All EKM groups showed complete DPPH· removal, indicating excellent antioxidant efficiency. However, there was no difference in antioxidant capacity between EKM1, EKM2 and EKM3. Subsequently, we validated the biocompatibility of EKM hydrogels using the CCK-8 assay to select the optimal EKM component. As shown in Supplementary Figure S3, EKM2 showed the best proliferation-promoting function for chondrocytes and BMSCs. Based on this data, EKM2 was selected as the Exos carrier (renamed EKM in subsequent studies).

### Preparation, cell uptake and biocompatibility of EKM hydrogels containing Exos (Exos@EKM)

**Figure 3.**
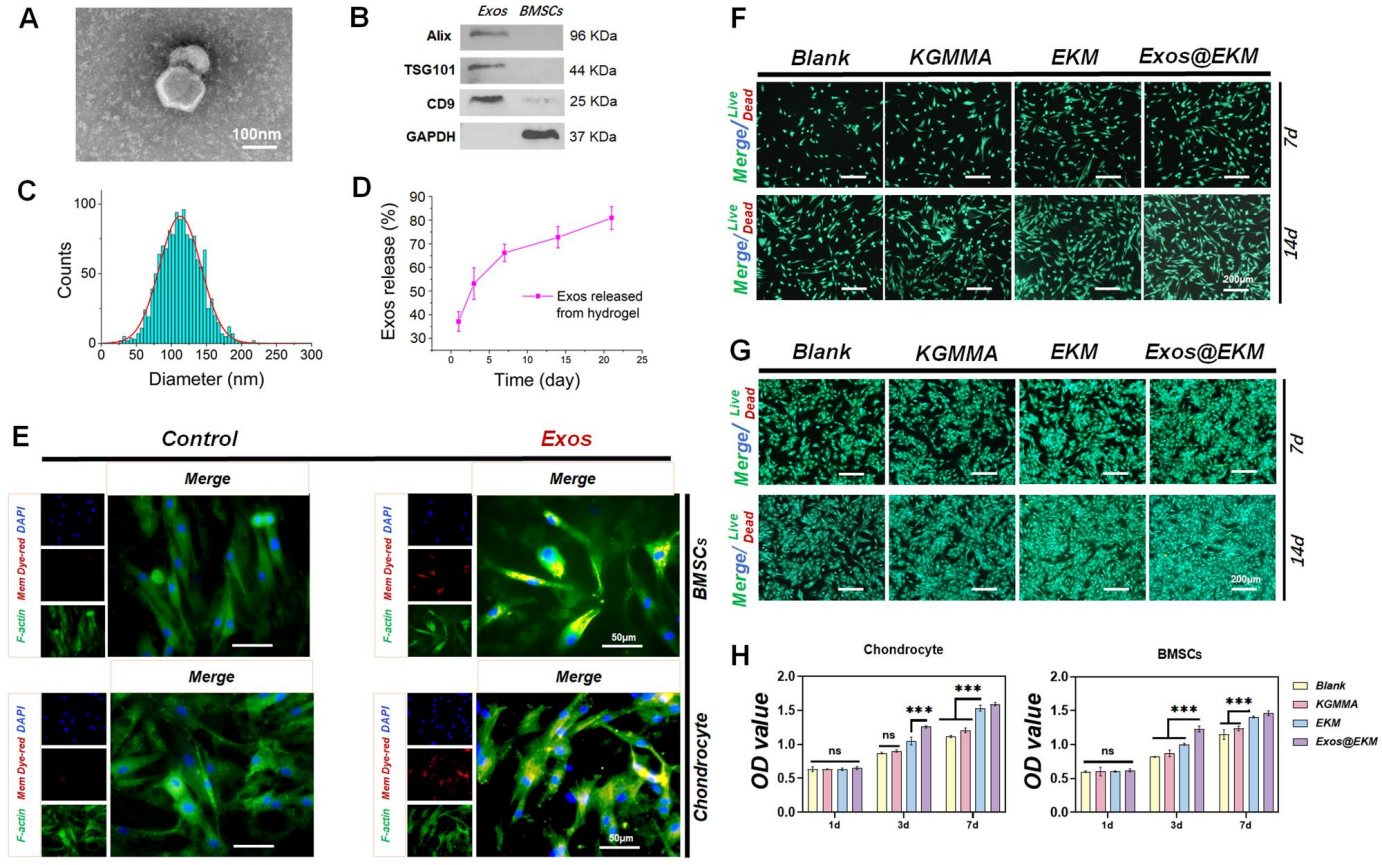
Isolation, identification and endocytosis of BMSC-derived Exos. (**A**) TEM image of Exos. (**B**) Western blotting for Alix, TSG101 and CD9 in BMSC-derived Exos. (**C**) NTA results of Exos. (**D**) Release curve of the Exos released from Exos@EKM. (**E**) After establishing Exos@EKM hydrogel, fluorescence image indicating the internalization of the released Exos from Exos@EKM by chondrocytes and BMSCs. Green fluorescence: cytoskeleton (F-actin); red fluorescence: Exos labeled with fluorescence; blue fluorescence: cell nucleus. (**F**)–(**H**) The biocompatibility of each group of hydrogels was subsequently validated. Including Live-Dead staining analysis of (**F**) chondrocytes and (**G**) BMSCs co-cultured with hydrogel, respectively, and (**H**) CCK-8 analysis. Data are presented as mean ± SD (*P < 0.05 or **P < 0.01 between the indicated groups).

The morphology of BMSC-derived Exos was observed using transmission electron microscopy (TEM), revealing a spherical microbubble-like structure (Figure 3A). Western blotting results (Figure 3B) demonstrated the presence of surface markers, such as Alix, TSG101 and CD9, confirming the successful isolation of BMSC-derived Exos. Nanoparticle tracking analysis (NTA) indicated an average size of 126.6 ± 47.3 nm for Exos, with 99.8% falling within the range of 30–200 nm (Figure 3C). These results demonstrated the successful isolation of BMSC-derived Exos. Subsequently, EKM hydrogels were loaded with varying concentrations of Exos (0 µg/ml, 50 µg/ml, 150 µg/ml, 250 µg/ml, 350 µg/ml, 500 µg/ml) for culturing chondrocytes, which were designated as 1-Exos@EKM, 2-Exos@EKM, 3-Exos@EKM, 4-Exos@EKM, 5-Exos@EKM and 6-Exos@EKM, respectively. The influence of different exosome concentrations on chondrocyte matrix secretion was assessed using Alcian Blue staining, while the viability and glycosaminoglycan (GAGs) content of chondrocytes were evaluated by measuring absorbance at 630 nm. Supplementary Figure S4 demonstrated that an exosome concentration of 250 µg/ml exerted the most significant effects on chondrocyte matrix secretion and cell viability. Consequently, this concentration (4-Exos@EKM) was selected for subsequent experiments and renamed Exos@EKM. Moreover, Exos@EKM hydrogels were constructed and cell uptake of released Exos was confirmed through Transwell assays using ExoSparkler-labeled Exos to visualize red fluorescence. ExoSparkler-labeled Exos were loaded into EKM hydrogel to establish Exos@EKM. Fluorescence imaging showed that both BMSCs and chondrocytes internalized Exos after the cells were co-cultured with Exos@EKM hydrogel for 24 h (Figure 3E). Quantitative analysis of exosome endocytic efficiency (Supplementary Figure S5) further confirmed that cells efficiently internalized a substantial quantity of Exos. Additionally, we characterized the release of Exos and EGCG encapsulated in the prepared hydrogel. As depicted in Figure 3D, approximately 76% of Exos were released within a period of 21 days. Additionally, the biocompatibility assessment of the prepared Exos@EKM hydrogel was conducted. The fluorescence images of live/dead staining also confirm satisfactory biocompatibility of the Exos@EKM hydrogel (Figure 3F and G). For both chondrocytes and BMSCs, majority cells exhibited green fluorescence indicative of live cells, with minimal red fluorescence observed for dead cells. The corresponding quantitative analysis further substantiated these observations (Supplementary Figures S6 and S7). The CCK-8 data demonstrated that the Exos@EKM hydrogel facilitated the proliferation of BMSCs and chondrocytes. As illustrated in Figure 3H, there was a significant disparity in cell viability between the group treated with Exos@EKM hydrogel and other groups after a culture period of 7 days, indicating that the hydrogel promotes BMSCs and chondrocytes proliferation through Exos release. In conclusion, it can be inferred that the Exos@EKM hydrogel demonstrates excellent biocompatibility while effectively regulating exosome release to influence cellular behavior.

### Exos@EKM enhances cartilage ECM metabolism in an oxidative stress microenvironment

**Figure 4.**
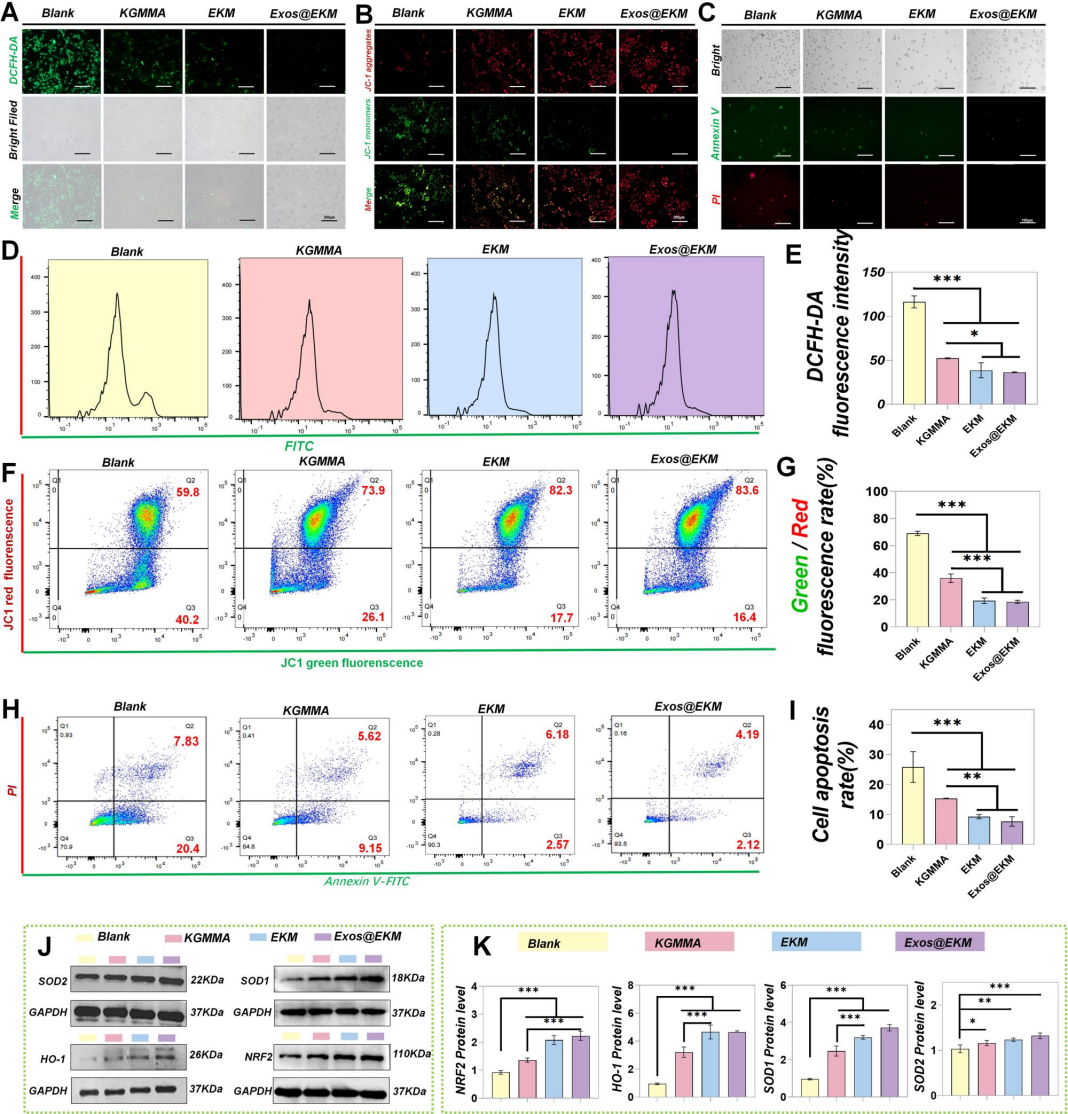
The protective effect of Exos@EKM hydrogel on chondrocytes cultured in oxidative stress microenvironment. (**A**)–(**C**) Fluorescence images of various probe-labeled chondrocytes, including: (**A**) cellular ROS fluorescent probe (DCFH-DA), (**B**) mitochondrial membrane potential fluorescent probe (JC-1) and (**C**) cellular apoptosis fluorescence staining (Annexin V-PI). (**D**)–(**I**) flow cytometry along with its associated quantitative analysis, including: (**D**) and (**E**) DCFH-DA staining, (**F**) and (**G**) JC-1 staining and (**H**) and (**I**) Annexin V-PI staining. (**J**) and (**K**) Additionally, Western blot analysis was presented to verify the effect of Exos@EKM on the expression level of NRF2 and its downstream related proteins within chondrocytes: (**J**) protein bands; (**K**) quantitative analysis of Western blot. Data are presented as mean ± SD (*P < 0.05, **P < 0.01 or ***P < 0.001 between the indicated groups).

This study confirmed the chondroprotective effect of Exos@EKM through *in vitro* experiments. Initially, the DCFH-DA assay demonstrated that KGMMA, EKM and Exos@EKM significantly reduced ROS levels in chondrocytes under hydrogen peroxide stimulation (Figure 4A). The results of JC-1 staining (Figure 4B) indicated that the hydrogels significantly mitigated mitochondrial depolarization induced by hydrogen peroxide stimulation, leading to a restoration of the mitochondrial membrane potential in the cells. Additionally, Annexin-PI staining demonstrated that these hydrogels effectively reduced apoptosis triggered by hydrogen peroxide exposure (Figure 4C), with the EKM hydrogel exhibiting the most pronounced protective effect on cell viability. This study further conducted quantitative analyses to elucidate the chondrocyte-protective properties of the hydrogels. Data from DCFH-DA-related flow cytometry indicated that EKM and Exos@EKM effectively mitigated oxidative stress in chondrocytes. The quantitative analysis substantiates this assertion (Figure 4D and E). Additionally, the Data of flow cytometry analysis of JC-1 staining revealed comparable trends, with both the EKM group and Exos@EKM group demonstrating optimal recovery levels of mitochondrial membrane potential (Figure 4F and G). This finding is in line with previous results, demonstrating the capacity of KGM and EGCG to efficiently eliminate surplus ROS within cells and mitigate oxidative stress. This antioxidant effect also influenced cell apoptosis, as depicted in Figure 4H and I, where co-cultured chondrocytes with Exos@EKM and EKM exhibited lower levels of cell apoptosis with no significant difference between the two groups. While KGMMA also showed an inhibitory effect on cell apoptosis, its combination with EGCG significantly enhanced EKM's antioxidant effect.

These findings suggest that EKM can eliminate ROS accumulation caused by cartilage injury and alleviate harsh oxidative stress microenvironments, thereby protecting chondrocytes at sites of cartilage damage. As a carrier for active ingredients, it can mitigate adverse effects on cell differentiation and induce tissue ingrowth within unfavorable microenvironments. Additionally, this study further investigated the potential mechanism by which Exos@EKM enhances cellular antioxidant capacity. Western Blot analysis revealed a significant increase in the levels of NRF2, SOD1, SOD2 and HO-1 in chondrocytes following co-culturing with gel samples from each group (Figure 4J and K). These findings suggest that Exos@EKM may activate the intrinsic antioxidant function of chondrocytes by modulating the NRF2-related signaling pathway and elevating cellular antioxidant enzyme levels.

**Figure 5.**
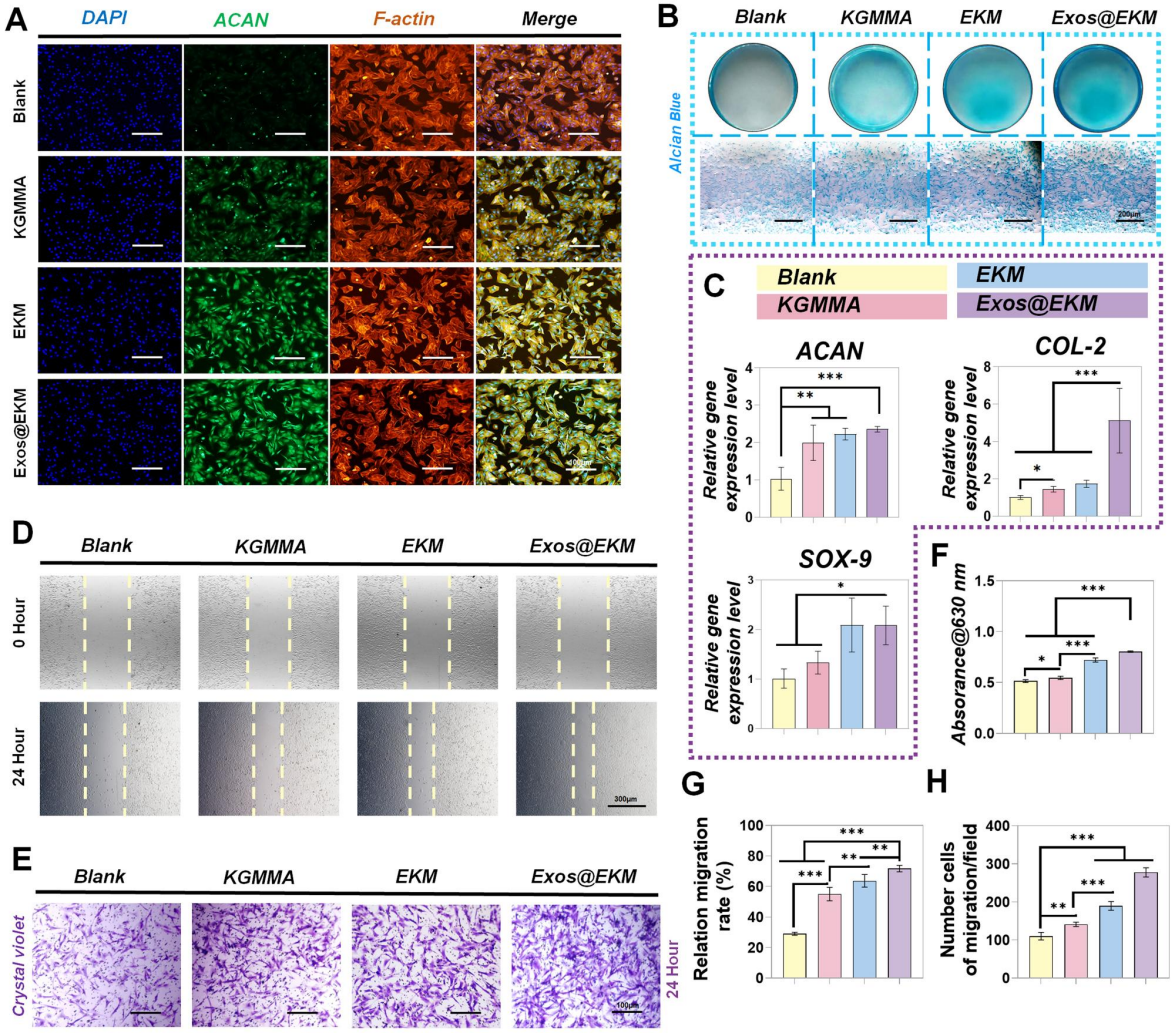
The capability of Exos@EKM hydrogel of enhancing cartilage ECM metabolism *in vitro*. (**A**) Immunofluorescence images demonstrate the increased production level and intensity of matrix (ACAN). (**B**) Representative images show enhanced anabolic markers of chondrocytes stained by Alcian blue staining. (**C**) Gene expression analysis using qPCR assay reveals upregulation of COL-2, ACAN and Sox9 (*n* = 4). (**D**, **E**) Representative images display improved cell migration as determined by crystal violet and scratch assays. (**F**) Quantitative assessment confirms the enhancement in Alcian blue staining analysis. (**G**, **H**) Quantitative assessment demonstrates improved cell migration as determined by crystal violet and scratch assays. Data are presented as mean ± SD (*P < 0.05, **P < 0.01 or ***P < 0.001 between the indicated groups).

Subsequently, we assessed the impact of hydrogel on cartilage ECM secretion under oxidative stress using a Transwell co-culture system. The fluorescent image in Figure 5A demonstrates that EKM increases the expression of ACAN (a characteristic protein for cartilage) in chondrocytes under oxidative stress conditions. Co-culturing cells with Exos@EKM results in optimal expression levels of characteristic proteins, as indicated by Alcian blue staining showing higher levels of glycosaminoglycans (GAG) compared to other groups (Figure 5B). This suggests that Exos@EKM can stimulate ECM secretion in chondrocytes under oxidative stress microenvironments. As shown in Figure 5C, Q-PCR results further support this finding, showing that chondrocytes co-cultured with Exos@EKM express the highest levels of specific cartilage genes Sox9, ACAN and COL-2. The released Exos play a crucial role by enhancing cell migration, proliferation and chondrogenic differentiation when internalized by endogenous chondrocytes and BMSCs. These Exos contain abundant miR-23a-3p which inhibits phosphatase and tensin homolog (PTEN) levels while enhancing protein kinase B (AKT) expression to promote cartilage regeneration [28, 39]. Our results demonstrate the synergistic effect of Exos and EKM with EKM acting as a carrier and microenvironment regulator. Concurrently, the hydrogel facilitated the restoration of mitochondrial membrane potential in cells, suggesting a recovery of cellular respiratory metabolic function, potentially attributable to the synergistic effects of KGMMA and EGCG. Based on our findings, it is evident that the released Exos promote chondrocyte proliferation, migration, redifferentiation ultimately leading to cartilage protection and regeneration. Moreover, our investigation delved into elucidating the potential safeguarding function of hydrogels to mitigate subchondral bone impairment. As depicted in Supplementary Figures S8–S10, it was observed that the hydrogel notably enhanced extracellular matrix secretion by BMSCs when subjected to hydrogen peroxide stimulation. The immunofluorescence staining images revealed that the Exos@EKM group demonstrated heightened levels of OPN protein and pertinent bone-related genes during osteogenic differentiation (Supplementary Figure S8). Furthermore, validation through alkaline phosphatase staining (ALP) and safranin red staining (ARS) corroborated these findings (Supplementary Figures S9 and S10). Collectively, these outcomes signify that Exos@EKM sustains BMSCs' differentiation potential under oxidative stress conditions, thereby implying a shielding effect of hydrogels on subchondral bone against cartilage damage.

### Exos@EKM activates the intrinsic antioxidant function in cartilage through the NRF2-ARE pathway

**Figure 6.**
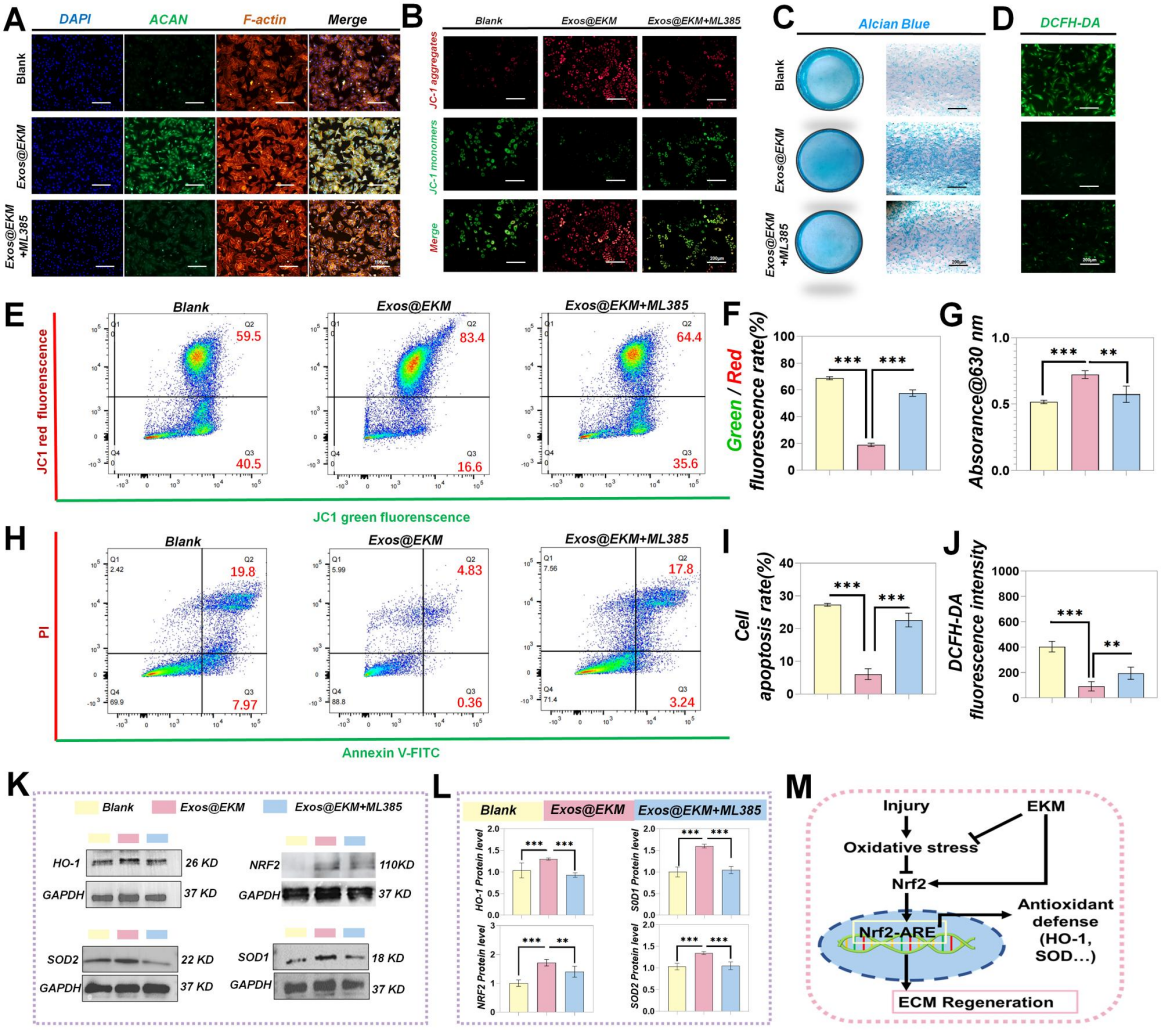
Exos@EKM hydrogels exhibited chondroprotective effects through the activation of the NRF2 pathway. (**A–I**) Following treatment with specific NRF2 inhibitors (ML385), images were obtained from (**A**) immunofluorescence labeling of ACAN, (**B**) JC-1 staining, (**C**) Alician blue staining and (**D**) cellular ROS levels labeled by DCFH-DA probe in chondrocytes. (**E**)–(**J**) Following treatment with ML385, the relevant flow cytometry and quantitative data were obtained, including: (**E**) flow cytometry data of JC-1 staining and (**F**) corresponding quantitative analysis, (**G**) quantitative data from Alcian blue staining, (**H**) flow cytometry data of Annexin V-PI staining and (**I**) corresponding quantitative analysis, as well as (**J**) quantitative data from DCFH-DA staining. (**K**, **L**) Western blot analysis demonstrates a reduction in the levels of NRF2 and its downstream proteins in chondrocytes, comprising (**K**) Western blot bands, (**L**) quantitative evaluation and (**M**) a schematic representation of the NRF2 Cascade implicated in EKM-mediated cartilage protection. Data are presented as mean ± SD (**P < 0.01 or ***P < 0.001 between the indicated groups).

In the preceding paragraph, it was demonstrated that hydrogel has the capacity to modulate the NRF2 signaling pathway in chondrocytes. To elucidate the pivotal role of the NRF2 pathway in regulating cellular antioxidant capacity and ECM secretion by Exos@EKM, chondrocytes were pre-treated with ML385, a specific inhibitor of NRF2. First, ML385 notably reduced the level of ACAN in chondrocytes (Figure 6A). The capacity of the hydrogel to inhibit mitochondrial depolarization in cells was also mitigated by ML385 (Figure 6B and E). Additionally, Alcian blue staining further confirmed the inhibition of Exos@EKM's promotion of ECM secretion in chondrocytes (Figure 6D and G). Although the hydrogel's capacity to scavenge ROS remained unaltered in the presence of the NRF2 inhibitor (Figure 6D and J), the results of flow cytometry revealed that the inhibition of NRF2 resulted in a diminished capacity of the hydrogel to shield cells from apoptosis (Figure 6H and I). The Western Blot results (Figure 6K and L) revealed that treatment with ML385 significantly decreased the level of NRF2 in cells in the Exos@EKM group, as well as the protein levels of SOD1, SOD2 and HO-1.

Therefore, these findings provide support for the role of Exos@EKM in enhancing endogenous antioxidant capacity through a NRF2-dependent pathway. Taken together, these findings indicate that the hydrogel-mediated protective effect on cartilage involves both endogenous and exogenous pathways. The exogenous pathway pertains to the inherent antioxidant properties of the hydrogel itself, which remain unaffected by NRF2 inhibition. Conversely, the NRF2 signaling pathway represents a primary endogenous antioxidant mechanism crucial for cartilage protection and significantly influences matrix secretion. The significance of NRF2 for cartilage regeneration lies in its ability to regulate antioxidant and anti-inflammatory defense proteins within cells by binding to specific antioxidant response elements (ARE) in the nucleus and protecting mitochondria. It is widely accepted that downregulation of KEAP1-dependent ubiquitination is a key mechanism for increasing NRF2 levels. In rheumatoid arthritis (RA), absence of NRF2 exacerbates oxidative stress and cartilage ECM degradation [40]. In the progression of osteoarthritis (OA), diminished levels of NRF2 also result in heightened production of metalloproteinases and pro-inflammatory factors, leading to degradation of the cartilage layer [41]. Several studies have demonstrated that during the early stages of OA, noncoding microRNA-146a-5p (miR-146a-5p) exacerbates inflammation-induced cartilage destruction by targeting the 3' untranslated region (3'UTR) and inhibiting NRF2 translation [42]. Furthermore, augmenting NRF2 levels in cartilage tissue is essential for enhancing therapeutic efficacy in OA. Current evidence suggests that KGM can reverse structural damage to the kidney and liver caused by a high-sugar microenvironment through modulation of NRF2 and NF-κB pathways, as well as alleviating oxidative stress and inflammation in type 2 diabetes rat models [43]. EGCG, the primary polyphenol extracted from green tea, exhibits potent antioxidant activity. Previous research has indicated that EGCG not only reduces reactive oxygen species (ROS) levels but also activates endogenous antioxidant pathways and downstream targets such as antioxidant proteins SLC7A11, HO-1 and GPX4 [44]. Currently, it has been confirmed that the EGCG-activated NRF2 pathway plays a pivotal role in the treatment of liver detoxification, intestinal inflammation and atherosclerosis [45–47]. Our findings demonstrate that EKM significantly upregulates NRF2 expression in chondrocytes as well as its downstream targets HO-1 and SOD enzyme levels. It is evident that within the oxidative stress microenvironment of cartilage damage, the synergistic activation of the NRF2 pathway by KGM and EGCG components of EKM initiates endogenous antioxidant action. Given the current lack of understanding regarding the mechanism of action of KGM-based biomaterials in cartilage repair, this study highlights the protective potential of the KGM-based biomaterial-NRF2-antioxidant axis for cartilage metabolism and provides preliminary evidence supporting NRF2 activation and subsequent endogenous antioxidant cascade as effective enhancers of ECM remodeling (Figure 6M).

### Anti-inflammatory properties of Exos@EKM

**Figure 7.**
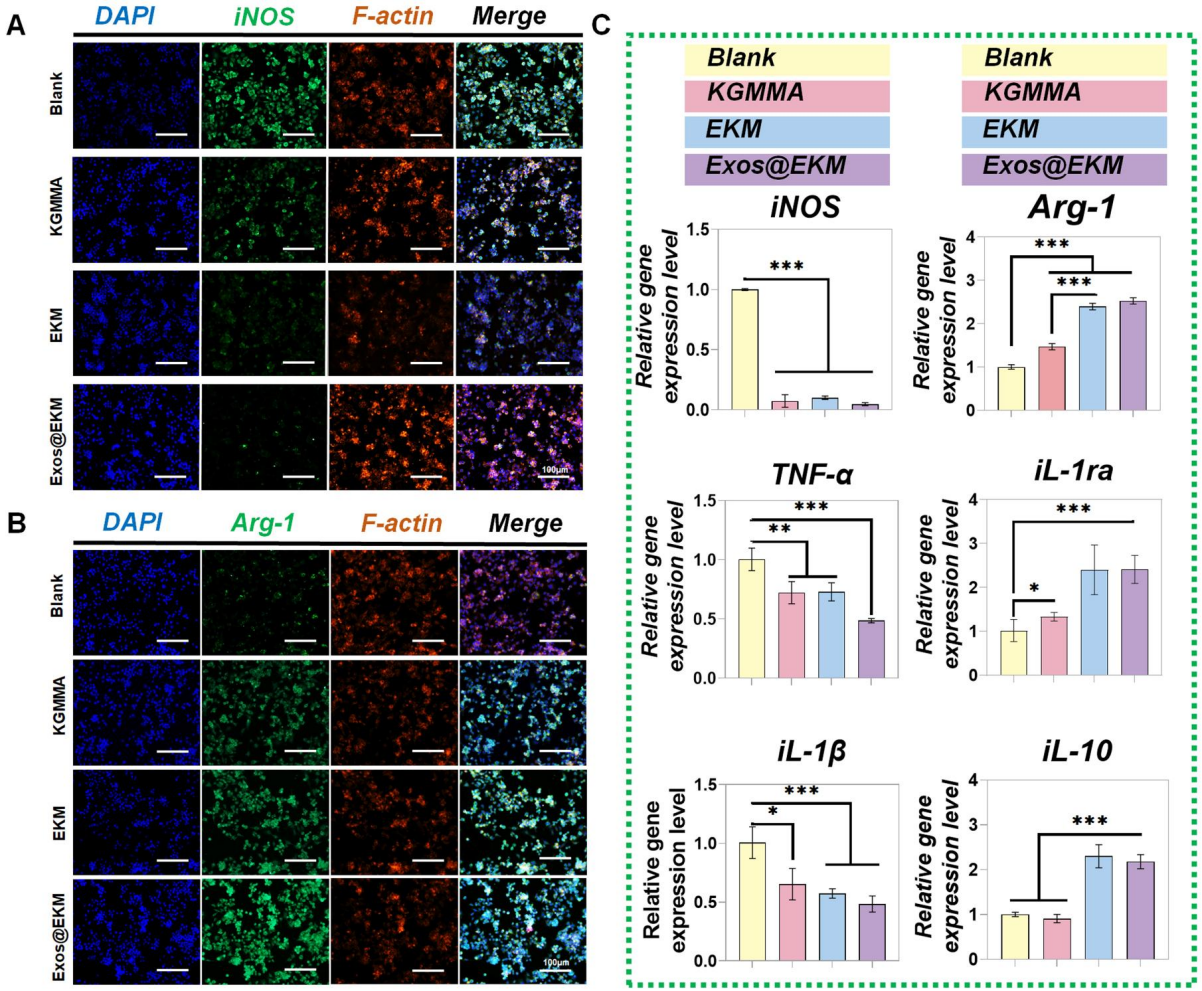
Exos@EKM hydrogel treatment on macrophage polarization and inflammation-related gene expression *in vitro*. (**A**, **B**) Fluorescence images showing iNOS (**A**) and Arg-1 (**B**) levels in RAW264.7 cells 48 h after LPS stimulation that co-cultured with the hydrogels. (**C**) qPCR evaluation of the expression of pro-inflammatory factors (IL-1β, TNF-α, iNOS) and anti-inflammatory factors (IL-10, Arg-1, IL-1ra) in RAW264.7 cells treated with different hydrogel scaffolds. Data are presented as mean ± SD (*P < 0.05, **P < 0.01 or ***P < 0.001 between the indicated groups).

Macrophages are the primary inflammatory mediators following cartilage damage and the polarization of macrophages to the pro-inflammatory M1 phenotype significantly contributes to local ROS production. As depicted in Figure 7, the impact of this konjac-derived hydrogel on RAW264.7 cells was investigated *in vitro*. Specifically, the expression of relevant inflammatory molecules was assessed using qPCR and immunofluorescence. In the qPCR analysis, the expression of M1 phenotype marker genes (IL-1β, TNF-α, iNOS) was effectively suppressed in the Exos@EKM group compared to the control group under LPS stimulation (Figure 7C). Conversely, there was an upregulation in the expression of M2 phenotype marker genes (IL-10, Arg-1, IL-1ra). Immunofluorescence results demonstrated lower levels of iNOS and higher levels of Arg-1 in macrophages from the Exos@EKM group (Figure 7A and B). These findings not only suggest that this hydrogel does not induce immune rejection but also exerts an immune-regulatory function by effectively inhibiting macrophage polarization towards M1 phenotype while promoting polarization towards M2 phenotype. Given that excessive ROS and M1/M2 imbalance contribute to a vicious cycle that is a major factor in microenvironment dysregulation, it is evident that this hydrogel design overcomes a significant obstacle to tissue regeneration and provides an optimal environment for recruiting, activating and efficiently repairing cartilage defects. The mechanism through which EKM regulates macrophage polarization may be associated with the interaction between the mannose units in KGM and the mannose receptors on macrophages, although the specific mechanism remains unclear [48]. This could serve as a potential direction for future research on KGM-based biomaterials.

### The role of Exos@EKM in promoting cartilage regeneration and joint function recovery *in vivo*

**Figure 8.**
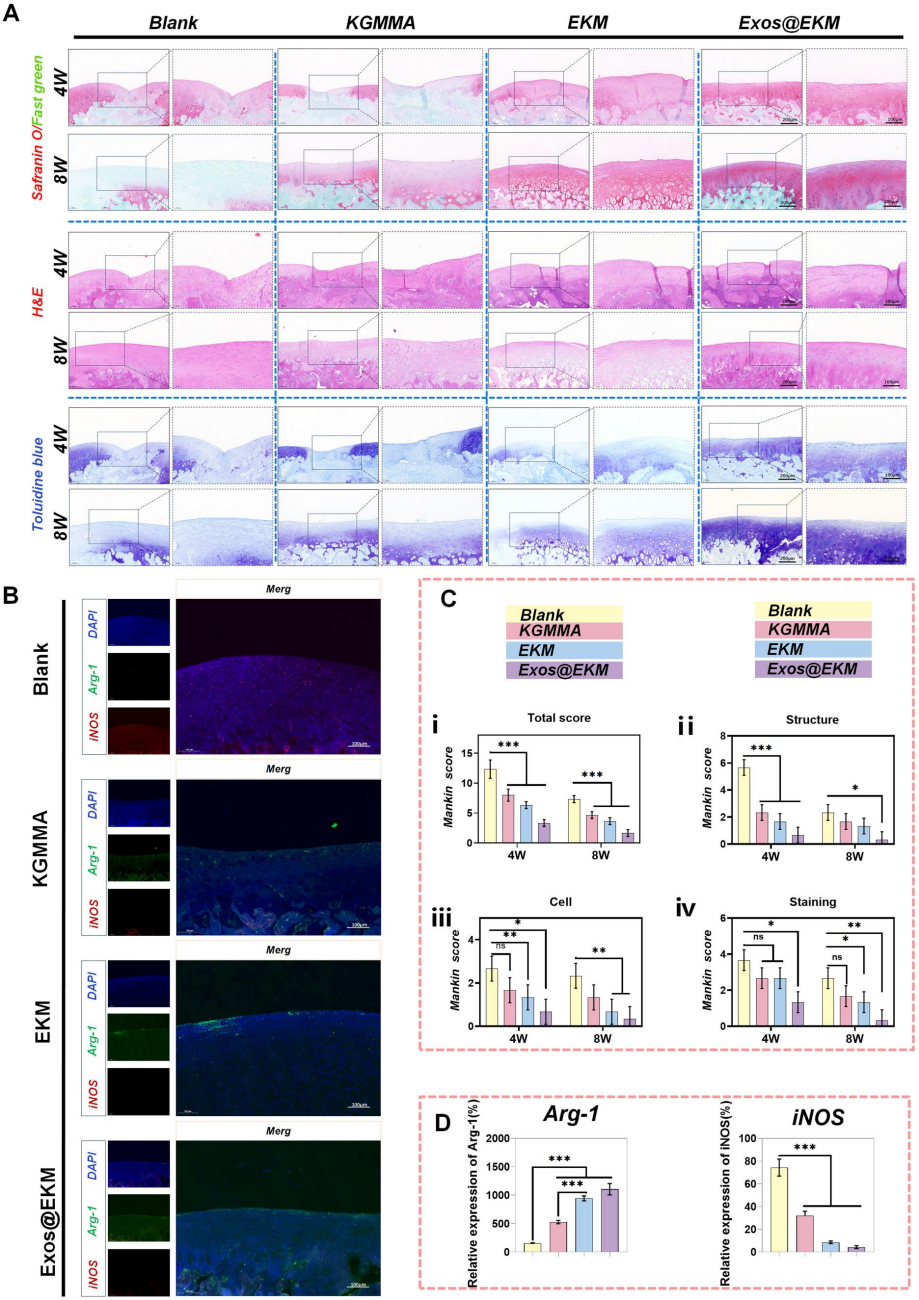
Exos@EKM hydrogels promoted articular cartilage regeneration in a rat full-thickness cartilage defect model. (**A**) Representative images of histology images of trochlear groove stained by hematoxylin and eosin (H&E). Sulfated glycosaminoglycans (GAGs) were detected by Safranin O (S.O.) and toluidine blue (T.B.) staining. The dotted boxes denoted the regenerated tissues. (**B**) Representative images of immunofluorescence staining for iNOS and Arg-1 in cartilage at 8 weeks postoperation. (**C**) Histological score of repaired cartilage using hydrogels based on a Mankin system (*n* = 4). (**D**) Quantitative analysis of fluorescent labeled iNOS and Arg-1 in cartilage at 8 weeks postoperation (*n* = 4). Data are presented as mean ± SD, *P < 0.05, **P < 0.01, ***P < 0.001.

In this study, a full-thickness cartilage defect was induced in rat models and subsequently filled with Exos@EKM hydrogel through *in situ* injection to assess the potential therapeutic effects of the konjac-derived hydrogel in promoting cartilage regeneration *in vivo*. Based on the results of SOF, TB and H&E staining (Figure 8A) at 4 weeks and 8 weeks post-treatment, the Exos@EKM group exhibited superior cartilage repair efficacy (the cartilage layer was stained red or blue, SOF: red; TB: blue). First, to investigate the *in vivo* retention characteristics of Exos in Exos@EKM, near-infrared fluorescence labeling (DiR labeling) was employed for exosome tracking. In this study, Exos@EKM was injected into the cartilage defect area of rat knees and it was observed that the Exos predominantly accumulated at the defect site of the knee joint. The results demonstrated that a significant local fluorescence signal remained detectable up to 4 weeks postinjection, indicating that the hydrogel carrier significantly enhanced the retention duration of Exos in the target area (Supplementary Figure S11). As the carrier of Exos, the EKM hydrogel integrated almost completely with the host tissue within 4 weeks postoperation without any inflammatory infiltration due to its excellent biocompatibility. Furthermore, at 4 weeks postsurgery, the Exos@EKM group exhibited the regeneration of a thinner and transparent cartilage layer. By 8 weeks, the transparent cartilage layer had thickened and the joint surface appeared smoother and flatter, with an intact subchondral bone structure. While the EKM group also displayed some degree of cartilage regeneration, it showed fewer mature chondrocytes and stained glycosaminoglycan components compared to the Exos@EKM group.

Immunofluorescence staining revealed that (Figure 8B), in the early postoperative period (within 4 weeks postoperatively), the Exos@EKM group demonstrated elevated levels of Arg-1 and reduced levels of iNOS compared to the control group. Quantitative data of immunofluorescence staining also confirms this (Figure 8D). These data indicating that Exos@EKM played an anti-inflammatory role in early cartilage injury *in vivo*. The results mentioned above were further validated by Mankin scores (Figure 8C). The untreated control group exhibited not only a higher level of inflammation, but also severe destruction of the joint surface and subchondral bone structure. While KGMMA demonstrated a certain anti-inflammatory and protective effect on the joint surface, it resulted in less chondrocyte-derived matrix production and mature chondrocytes were difficult to observe in the defective area.

**Figure 9.**
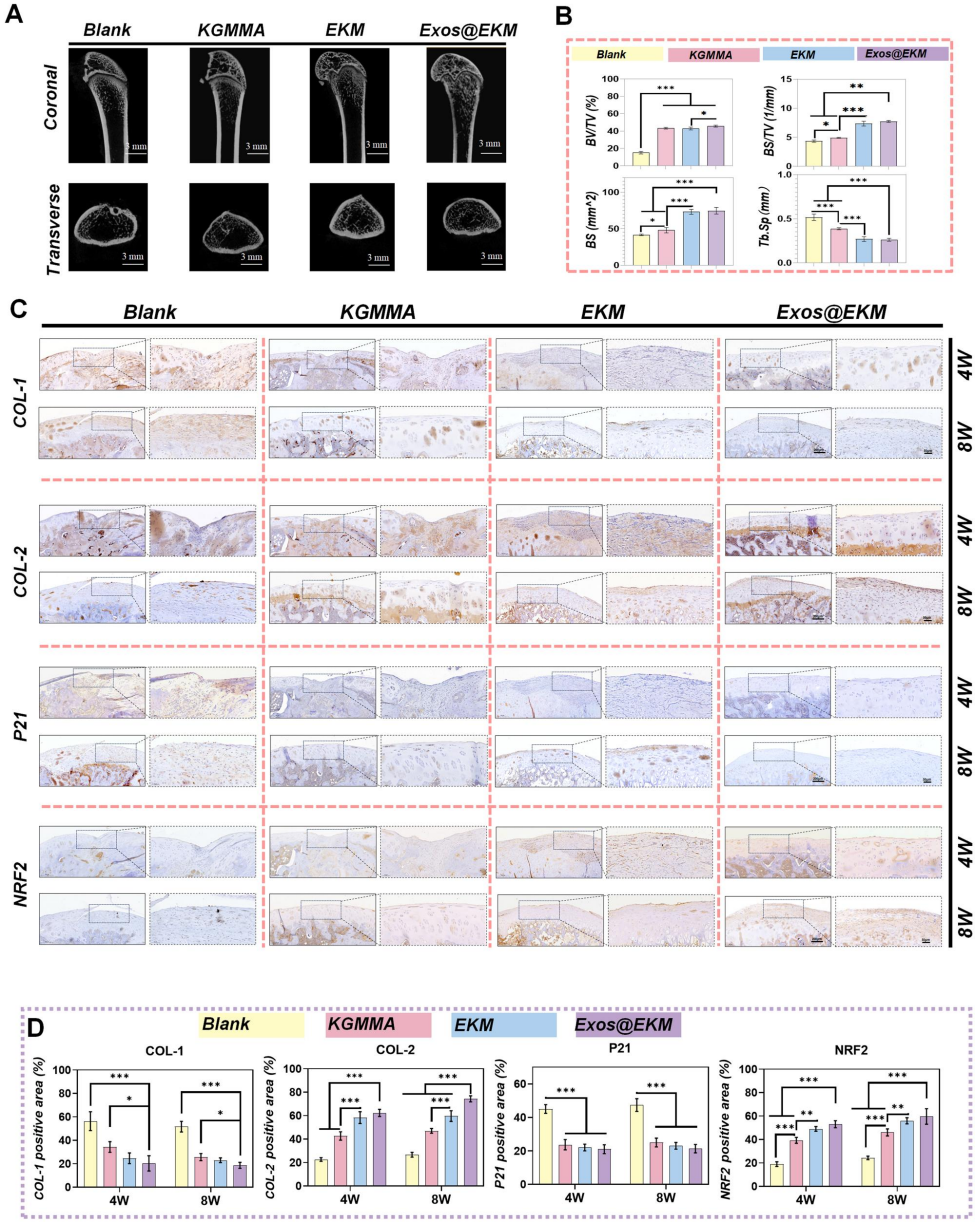
Exos@EKM hydrogel enhanced cartilage regeneration and restored motor function of knee via activating the endogenous antioxidant pathway mediated by NRF2. (**A**) Representative images Micro-CT of the knee joint at 8 weeks postoperation. (**B**) Quantitative analysis of the bone morphometric bone volume (BV) versus tissue volume (TV) value (*n* = 4). (**C**) Representative images of immunohistochemical (IHC) analysis of trochlear groove stained by COL-1, COL-2, P21 and NRF2. The dotted boxes denoted the regenerated tissues. (**D**) Quantitative analysis of the IHC stained areas for COL-1, COL-2, NRF2 and P21 (*n* = 4). Data are presented as mean ± SD (*P < 0.05 or **P < 0.01 between the indicated groups).

Additionally, MicroCT results confirmed the protective role of the hydrogel on early cartilage injury, as depicted in Figure 9A and B. In contrast to the untreated control group which showed severe joint tissue loss and disruption of subchondral bone structure integrity, Exos@EKM displayed a smooth and flat cartilage layer with intact subchondral bone structure. On the front of ECM rebuilding, the Exos@EKM group displays a harmonious expression of collagen, as evidenced by an increased level of hyaline cartilage marker COL-2 and decreased level of fibrous cartilage marker COL-1 (Figure 9C and D). Importantly, the Exos@EKM hydrogels elicited endogenous NRF2 pathway activation *in vivo*, as evidenced by elevated expression of NRF2 (Figure 9C and D). Accordingly, the hydrogel significantly inhibited the level of oxidative DNA damage marker p21, indicating a rebalanced antioxidant/pro-oxidant equilibrium for local ROS damage (Figure 9C and D).

**Figure 10.**
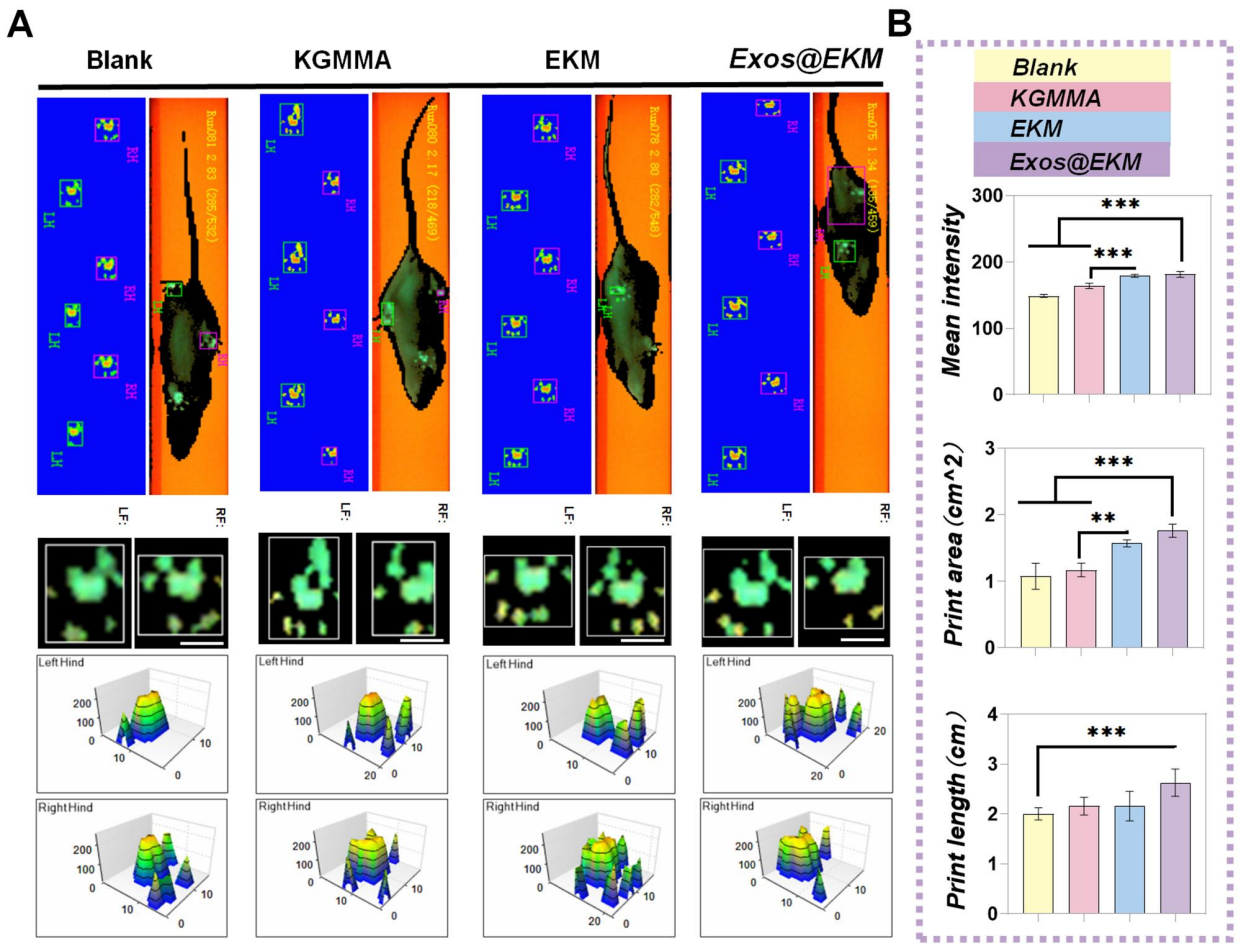
Footprint analysis of rat models at 8 weeks postsurgery. (**A**) The morphology, length and force intensity of rat footprints; (**B**) Statistical data on footprint morphology and average intensity. Bar = 1 cm. (*n* = 4, *P < 0.05, **P < 0.01, ***P < 0.001).

Furthermore, rat pawprint analysis provided further confirmation of Exos@EKM's capbility to restore knee joint function (Figure 10). According to the quantitative data, the rats in the Exos@EKM group showed a larger and more evenly distributed force area under the hindpaw print, indicating the best recovery of knee joint function in rats. In recent years, natural biocompatible substances have emerged as a promising direction for designing biomaterials for cartilage repair due to their intrinsic bioactivity that allows for cell adhesion, proliferation or regulation of cell behavior through autologous cell mobilization [22]. Given the heterogeneous components of cartilage ECM and the presence of oxidative stress/inflammatory microenvironment after injury, careful design is required when applying natural polysaccharides to cartilage tissue engineering. This study revealed that KGM's intrinsic antioxidant properties along with its binding with EGCG create an ideal environment for chondrocyte adhesion and proliferation necessary for recruiting adjacent stem cells for tissue reconstruction. On one hand, based on KGM's bioactivity and EGCG binding capacity, this hydrogel plays a protective role for cartilage through both direct removal of ROS and activation of endogenous antioxidant pathways dependent on NRF2 pathway. Thus, creating an oxidation-reduction friendly microenvironment conducive to tissue repair and regeneration. On the other hand, following modification with MA (i.e. KGMMA), relying on its good gel properties and porous structure makes it an ideal carrier for encapsulating Exos enabling them to fully function as extraordinary ECM-driven agents in repairing damaged cartilage within an oxidative stress microenvironment.

## Conclusion

In conclusion, the intricate interplay between excessive ROS and uncontrolled inflammation establishes a detrimental feedback loop that not only impedes cartilage repair but also accelerates degenerative pathological changes. This cycle may significantly compromise the efficacy of loading Exos into scaffolds for treating cartilage defects. To address these challenges, we developed a natural konjac-derived Exos@EKM injectable hydrogel, capitalizing on the unique advantages of KGM through its “clearing-activating-delivering-regulating” four-dimensional linkage mechanism. This hydrogel not only forms an *in situ* cartilage regeneration scaffold via UV curing but also effectively mitigates oxidative stress by eliminating excessive ROS through KGM and EGCG. Simultaneously, it activates the NRF2-dependent intrinsic antioxidant capacity within cells. Specifically, KGM exhibits a dual antioxidant mechanism: it directly scavenges free radicals while enhancing the expression of endogenous antioxidant enzymes such as SOD1 and heme HO-1. Notably, the exogenous antioxidant activity of KGM is significantly amplified when conjugated with EGCG. Furthermore, the methacryloyl-modified hydrogel demonstrates excellent adaptability to irregular cartilage defects, enabling rapid *in situ* crosslinking and providing robust mechanical stability and strength. These properties make it a highly promising candidate for tissue engineering scaffolds. As a multidimensional biologically synergistic carrier, the porous structure and high density of functional groups in KGM facilitate efficient exosome loading and sustained release, thereby promoting cartilage regeneration. Additionally, the hydrogen bonding between KGM and EGCG enhances both material stability and antioxidant properties. The hydrogel also exhibits potent immunomodulatory effects, protecting chondrocytes in inflammatory microenvironments by promoting macrophage polarization toward an anti-inflammatory M2 phenotype. This process suppresses pro-inflammatory factors such as iNOS and IL-1β, while enhancing the production of anti-inflammatory mediators like Arg-1 and IL-10. By disrupting the vicious cycle between oxidative stress and exacerbated inflammation, the hydrogel safeguards residual cartilage cells, fosters a conducive environment for cell proliferation, recruitment and chondrogenic differentiation and ultimately achieves transparent cartilage regeneration while preserving subchondral bone integrity. Compared with the widely studied gelatin methacrylate (GelMA) and hyaluronic acid (HA) hydrogels, which exhibit relatively limited antioxidant and anti-inflammatory capabilities, EKM demonstrates significant antioxidant properties, as evidenced by the Supplementary Figure S12. The uniqueness of EKM stems from its natural polysaccharide-polyphenol complex system, where the synergistic interaction between konjac glucomannan (KGM) and epigallocatechin gallate (EGCG) not only ensures physical stability but also actively remodels the microenvironment of damaged tissues through NRF2 signaling pathway activation and macrophage reprogramming toward an anti-inflammatory phenotype. This multi-dimensional interaction mechanism, encompassing “scavenging-activating-delivering-regulating,” cannot be achieved by single-component materials such as GelMA, which primarily provides mechanical support, or HA, whose functionality depends on passive degradation. Furthermore, the degradation products of EKM, including mannose units, may enhance its anti-inflammatory effects by interacting with mannose receptors on macrophage surfaces. In summary, this innovative KGM-based hydrogel, characterized by its robust antioxidant properties, anti-inflammatory effects, chondrogenic activities and injectable nature, holds great promise for clinical translation. Moreover, it provides novel insights into the utilization of natural plant polysaccharides in tissue engineering applications.

## Supplementary Material

rbaf060_Supplementary_Data

## Data Availability

The raw data and processed data required to reproduce these findings are available from the corresponding author upon request.
