# Supplementary material for: Injectable exosome-reinforced konjac glucomannan composite hydrogel for repairing cartilage defect: activation of endogenous antioxidant pathways
Source: Regen Biomater. 2025 Jun 17;12:rbaf060. doi: 10.1093/rb/rbaf060 (PMC12311292; doi:10.1093/rb/rbaf060)
Supplement: rbaf060_Supplementary_Data [file rbaf060_supplementary_data.zip › Supporting Information.docx]

**Supporting Information.**

**Injectable exosome-reinforced konjac glucomannan composite hydrogel for repairing cartilage defect: activation of endogenous antioxidant pathways**

*Cong Ye ^1,^*^#^*, Jiabao Xu ^1,^*^#^*, Youjian Wang ^1^, Minrui Ji ^1^, Ran Tao ^1,^* , Fei Han ^1,^* and Peng Zhou ^2,^*.*

^1^ Department of Orthopaedics, Affiliated Hospital of Nantong University, Medical School of Nantong University, Nantong 226001, China.

^2^ Department of Sports Medicine, The 941th Hospital of Joint Logistic Support Force of Chinese People’s Liberation Army, Xining 810000, China.

^#^ *These authors contributed equally to this work.*

*Corresponding author.

Email: [zhoupeng7seven@outlook.com](mailto:zhoupeng7seven@outlook.com) (P.Z.); feyhan50089@outlook.com (F.H.); [nigetr007@163.com](mailto:trniger007@163.com) (R. T.)

**Figures:**

**
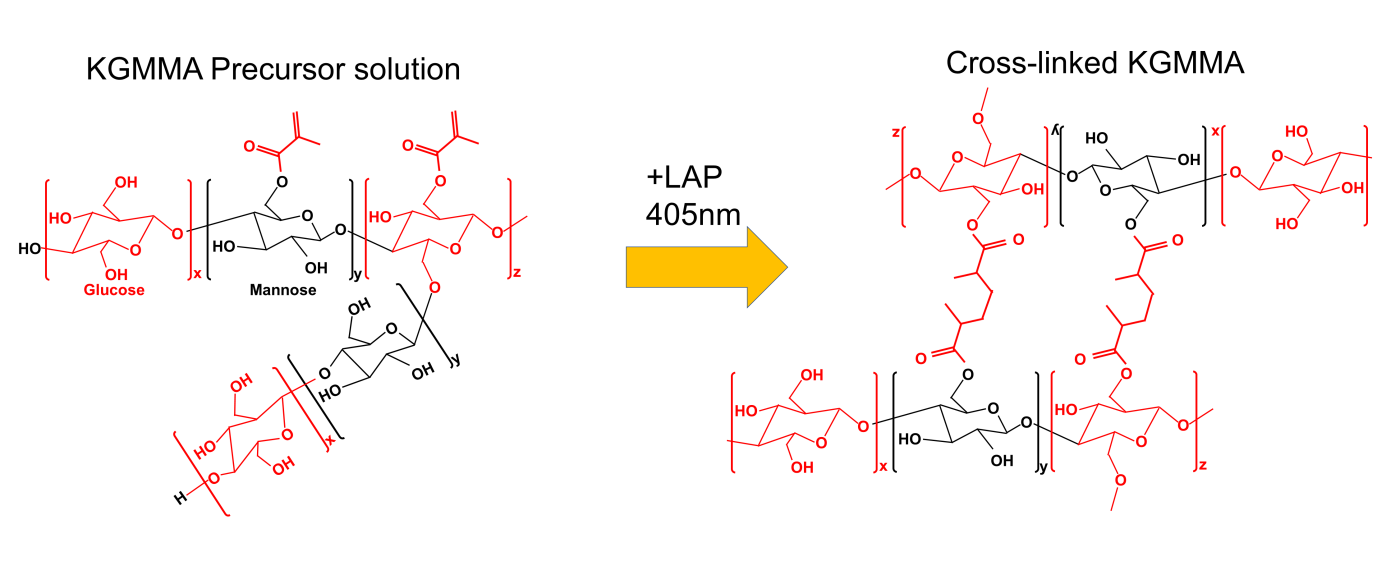
**

**Figure S1.** Molecular formula of the hydrogel crosslinking induced by UV.


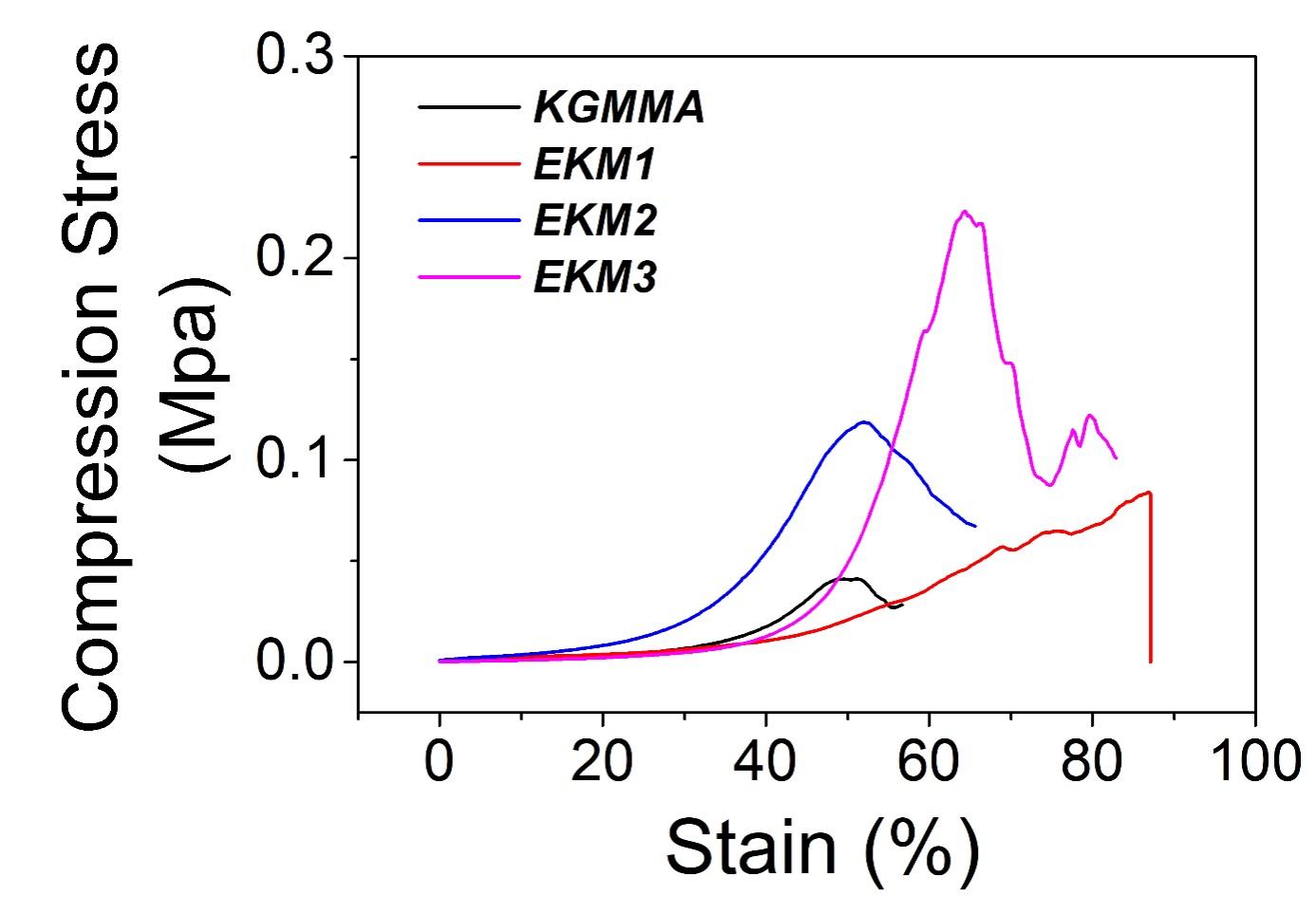


**Figure S2.** Stress strain curve of each group of hydrogels obtained from compression stress test.


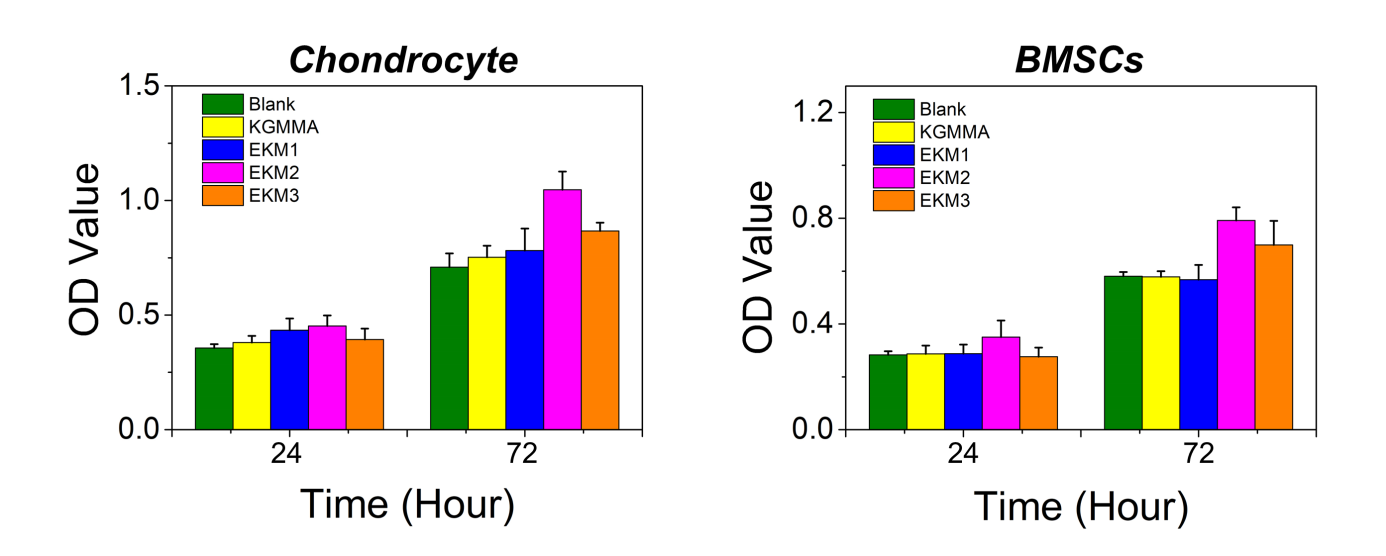


**Figure S3.** CCK-8 analysis demonstrated the biocompatibility of each EKM hydrogel group following co-culture with chondrocytes and BMSCs.


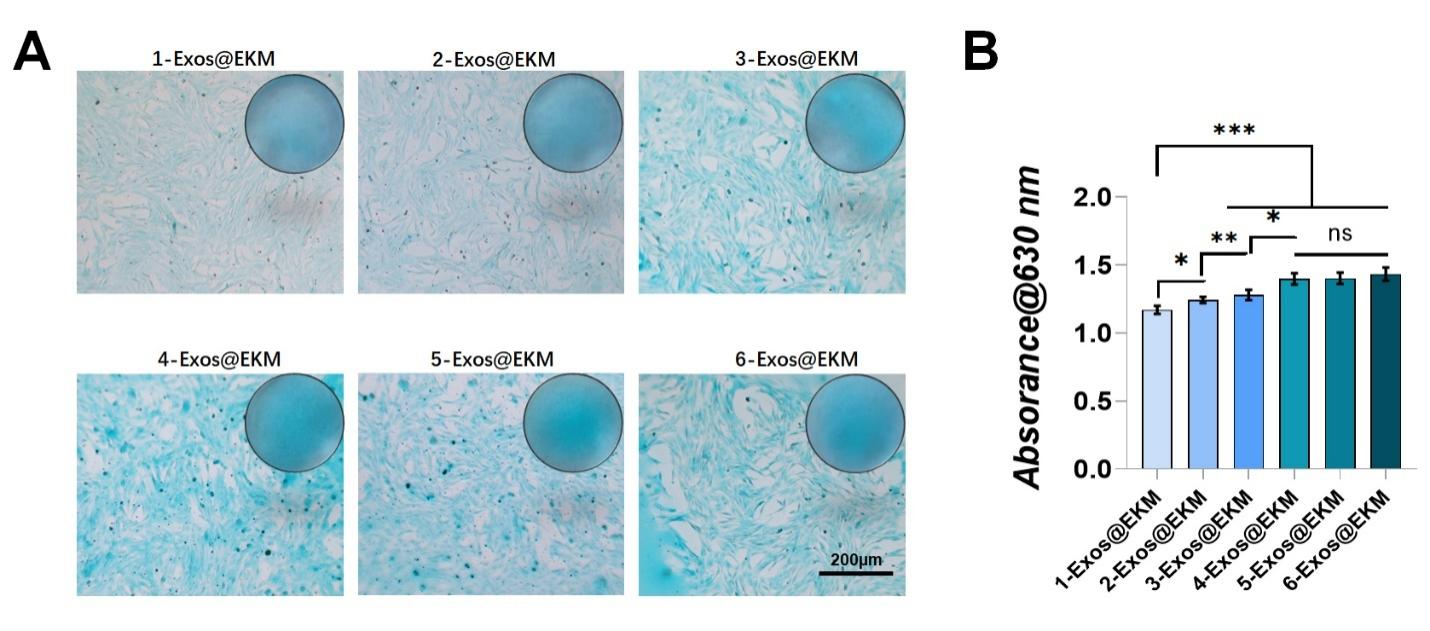


**Figure S4.** The influence of different Exos concentrations on chondrocyte matrix secretion was assessed using Alcian Blue staining. (A) Representative images of Alcian Blue staining. (B) Quantitative analysis of GAGs by measuring absorbance at 630 nm (n = 5). Data are presented as mean ± SD (*P < 0.05 or **P < 0.01 between the indicated groups).


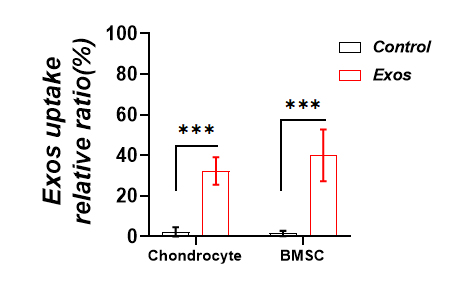


**Figure S5.** Quantitative analysis of exosome endocytosis efficiency (n = 4). Data are presented as mean ± SD (*P < 0.05 or **P < 0.01 between the indicated groups).


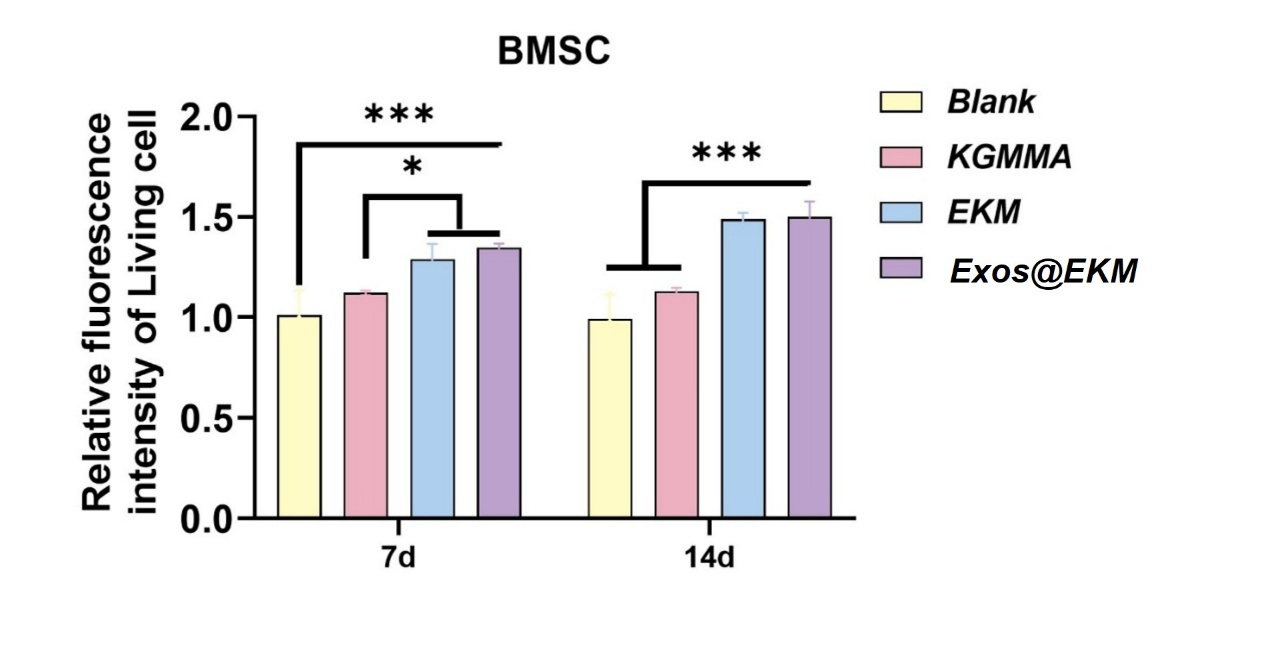


**Figure S6.** Quantitative analysis of BMSCs live-dead staining (n = 5). Data are presented as mean ± SD (*P < 0.05 or **P < 0.01 between the indicated groups).


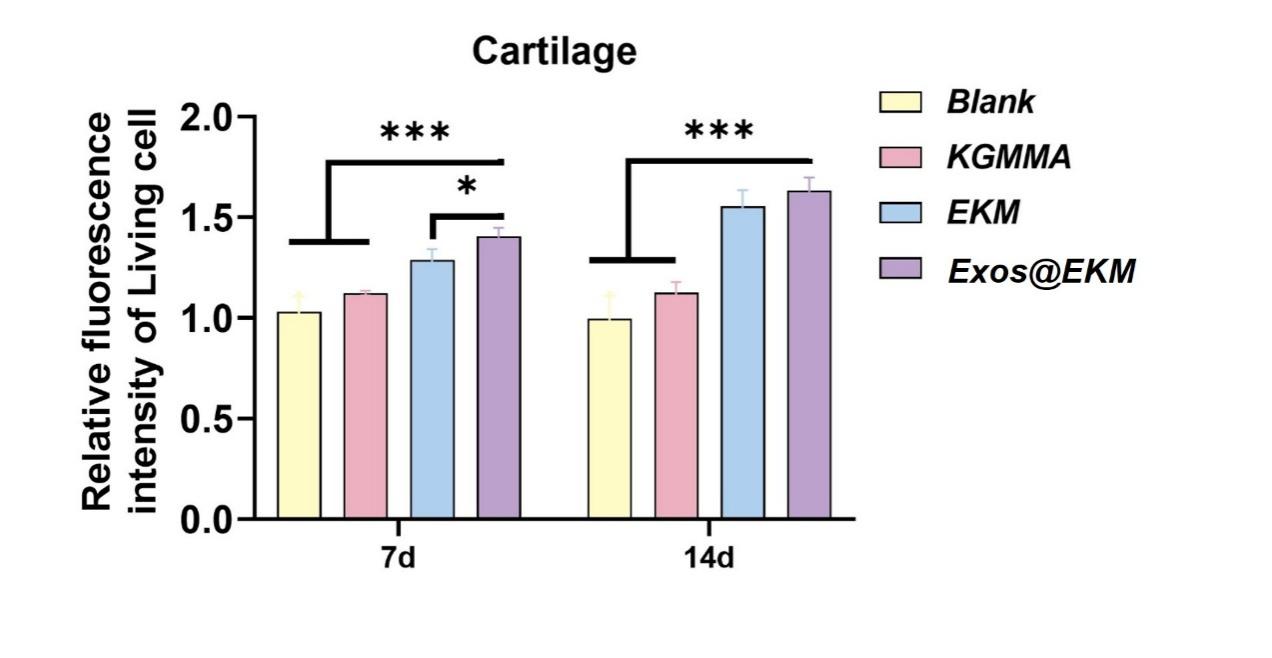


**Figure S7.** Quantitative analysis of chondrocytes live-dead staining (n = 5). Data are presented as mean ± SD (*P < 0.05 or **P < 0.01 between the indicated groups).


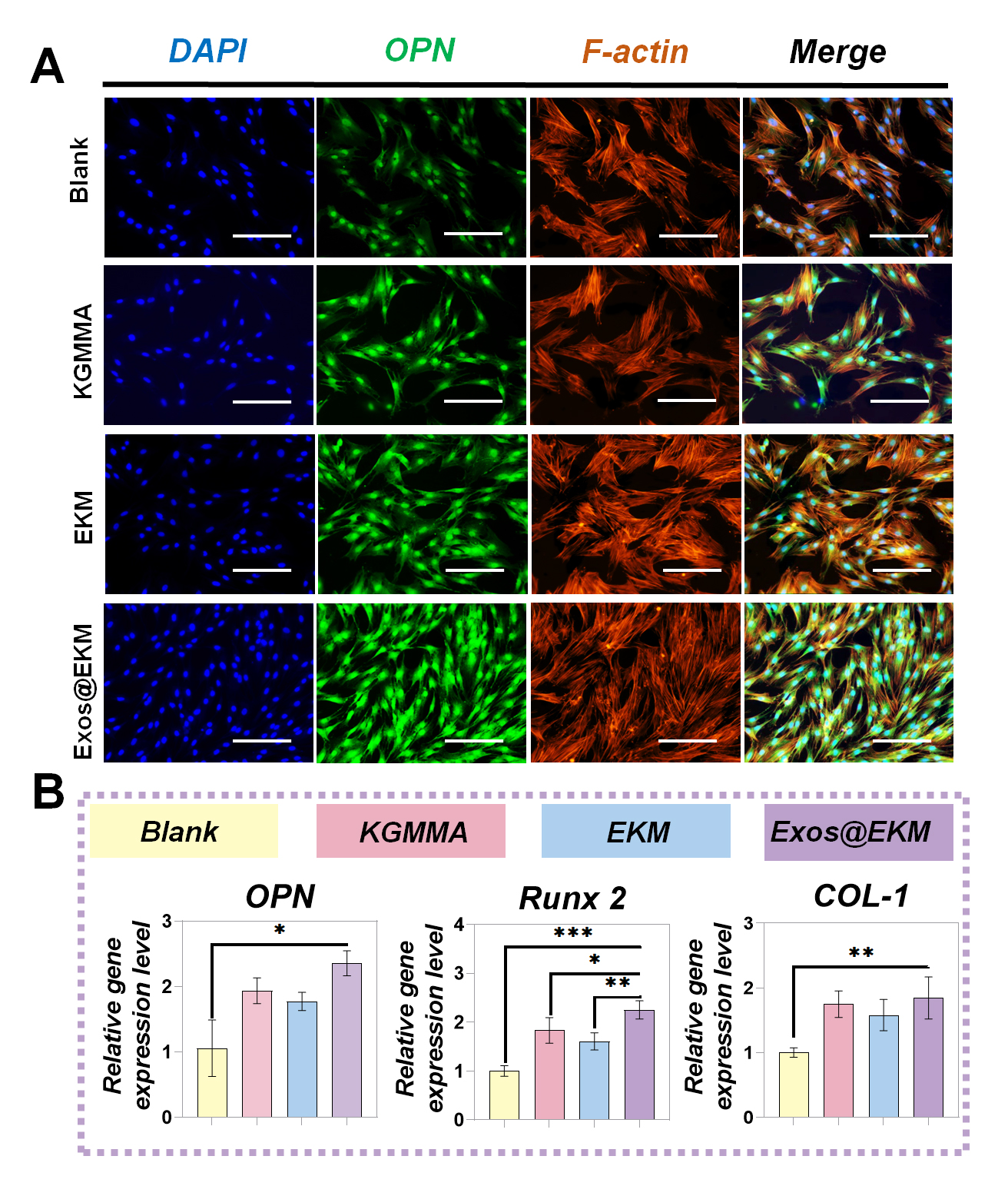


**Figure S8.** The capability of Exos@EKM hydrogel of enhancing osteogenic metabolism of BMSCs oxidative stress microenvironment. (A) Immunofluorescence images demonstrate the increased production level and intensity of matrix (OPN). (E) Gene expression analysis using Q-PCR assay reveals upregulation of osteogenic differentiation-related genes (n = 4). Data are presented as mean ± SD (*P < 0.05 or **P < 0.01 between the indicated groups).


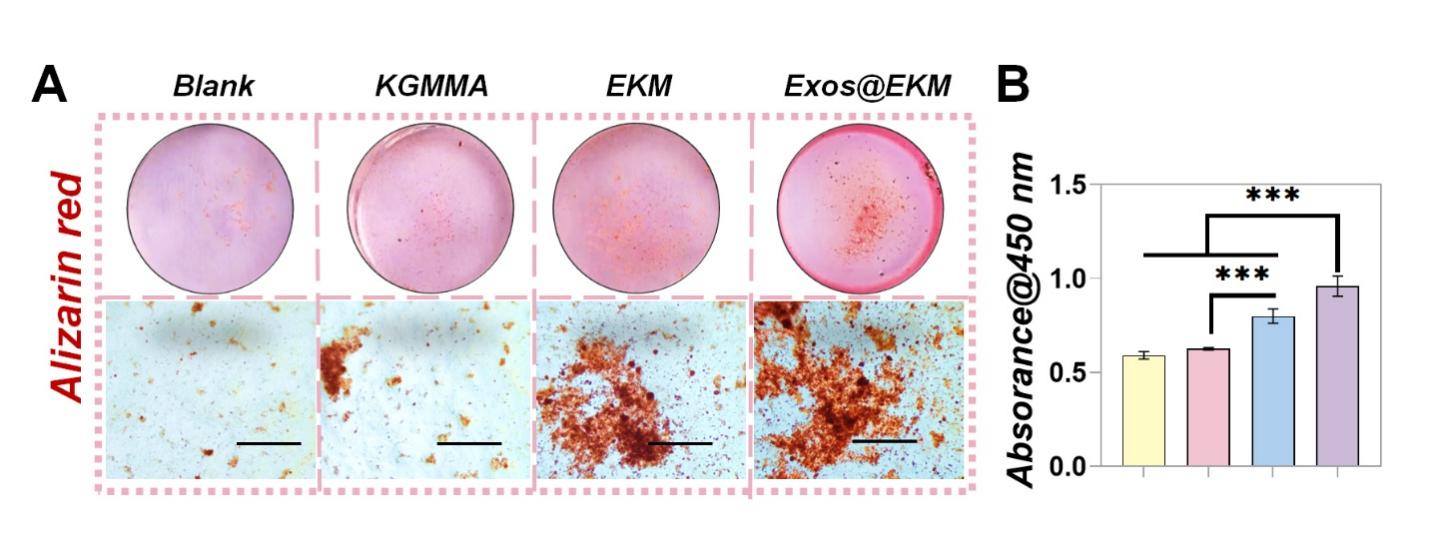


**Figure S9.** (A) The representative images of alizarin red staining (ARS) and (B) its subsequent quantitative analysis. (n = 4). Data are presented as mean ± SD (*P < 0.05 or **P < 0.01 between the indicated groups).


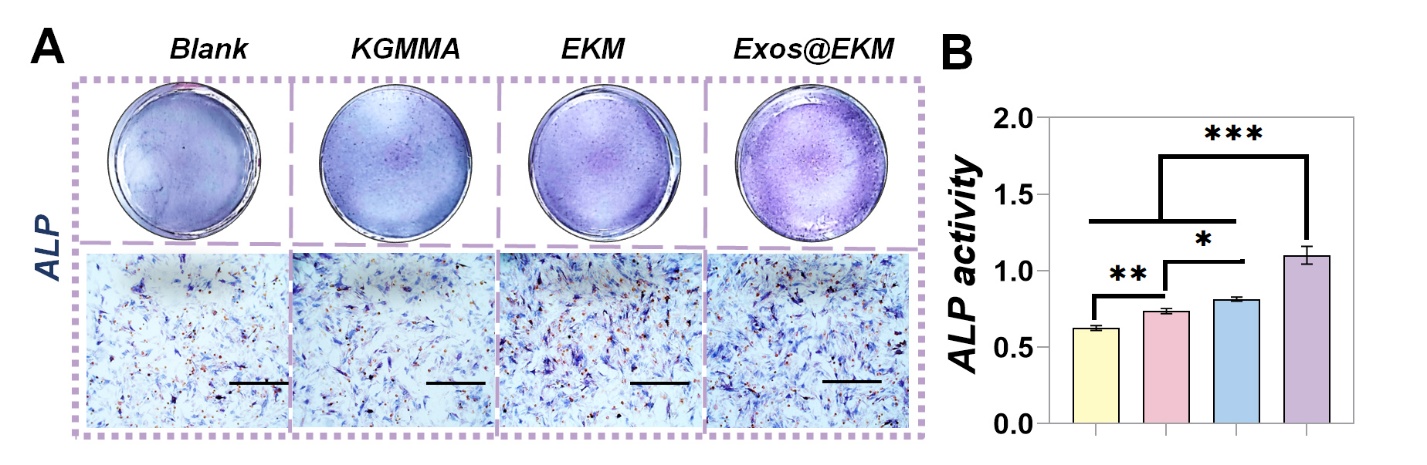


**Figure S10.** (A) The representative images of alkaline phosphatase (ALP) staining and (B) its subsequent quantitative analysis. (n = 4). Data are presented as mean ± SD (*P < 0.05 or **P < 0.01 between the indicated groups).


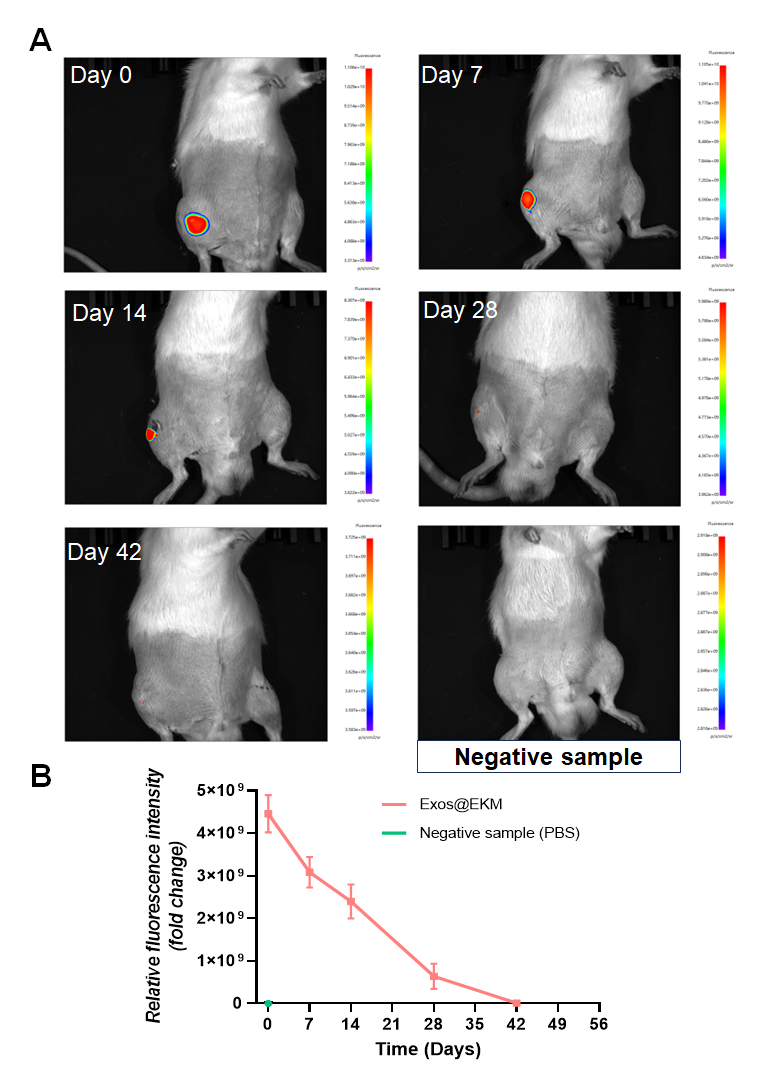


**Figure S11.** (A) Representative in vivo fluorescence intensity images of hydrogels. (B) Changes in fluorescence of hydrogels implanted in rat, as measured by in vivo imaging system. Data are presented as mean values ± SD, with n = 3. P > 0.05 (two-tailed Student's t-test).


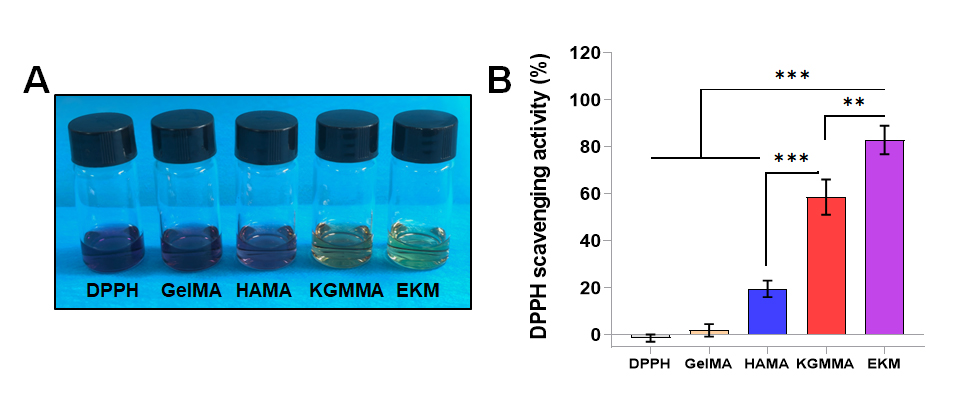


**Figure S12.** (A) Digital images showing the color changes of each hydrogel group after reacting with 2,2-diphenyl-1-picrylhydrazyl (DPPH). (B) Quantitative analysis of DPPH radical scavenging efficiency for each hydrogel group. Data are presented as mean ± SD, with n = 5. *p < 0.05, **p < 0.01, ***p < 0.001.

**Table S1.** The antibody used in WB and IF of this study.

|  | PRODUCT | Manufacturer | Catalog | Applications |
| --- | --- | --- | --- | --- |
| Primary antibody for WB | NRF2 | Proteintech | No. 16396-1-AP | WB |
|  | SOD1 | Proteintech | No. 10269-1-AP | WB |
|  | SOD2 | Proteintech | No. 24127-1-AP | WB |
|  | HO-1 | Proteintech | No. 10701-1-AP | WB |
|  | β-Tubulin | Proteintech | No. 10068-1-AP | WB |
|  | GAPDH | Proteintech | No. 10494-1-AP | WB |
| Secondary Antibody for WB | Multi-rAb HRP-Goat Anti-Rabbit Recombinant Secondary Antibody (H+L) | Proteintech | No. RGAR001 | WB |
| Primary antibody for IF | Aggrecan(ACAN) | Affinity | DF7561 | IF |
|  | OPN | Proteintech | No. 22952-1-AP | IF |
|  | Arginase-1(Arg-1) | Proteintech | No. 16001-1-AP | IF |
|  | iNOS | Proteintech | No. 80517-1-RR | IF |
| Secondary antibody for WB | CoraLite488-conjugated Goat Anti-Rabbit IgG(H+L) | Proteintech | No. SA00013-2 | IF |

**Table S2.** The primer sequences used in the Q-PCR of this study.

| Chondrocytes and BMSCs | | |
| --- | --- | --- |
| Gene |  | Primer sequence |
| Sox 9 | Forward | GGAGCTCGAAACTGACTGGAA |
|  | Reverse | GAGGCGAATTGGAGGAGGA |
| ACAN | Forward | CTGGGTGGATGCAGAGAGAC |
|  | Reverse | TTGGTTTGGACGCCACTTCT |
| COL-2 | Forward | GAGAACCTGGTACCCCTGGA |
|  | Reverse | CCTTATGACTCCCATCTG |
| GAPDH | Forward | CCGCATCTTCTTGTGCAGTG |
|  | Reverse | CCGATACGGCCAAATCCGTT |
| Runx2 | Forward | CCAACTTCCTGTGCTCCGTG |
|  | Reverse | GTGAAACTCTTGCCTCGTCCG |
| Opn | Forward | GCAGGACTGAAGGAGC |
|  | Reverse | GAGACAGGAGGCAAGG |
| Col I | Forward | TGGATGGCTGCACGAGT |
|  | Reverse | TTGGGATGGAGGGAGTTTA |
| Raw 264.7 | | |
| Gene |  | Primer sequence |
| iL-1β | Forward | CTACCTGTGTCTTTCCCGTG |
|  | Reverse | TTTGTTGTTCATCTCGGAGC |
| Arg-1 | Forward | ATCAACACTCCCCTGACAACC |
|  | Reverse | TCGCAAGCCAATGTACACGAT |
| iNOS | Forward | ACGCTTCACTTCCAATGCAAC |
|  | Reverse | CAGCCTCATGGTAAACACGTTC |
| iL-10 | Forward | GAGAAGCATGGCCCAGAAATC |
|  | Reverse | GAGAAATCGATGACAGCGCC |
| iL-1ra | Forward | AGAGCCCCTTATAGTCACGAA |
|  | Reverse | TACACCCTGCAAAAGTTGTTCC |
| TNF-α | Forward | CTGTAGCCCACGTCGTAGCAA |
|  | Reverse | TGTCTTTGAGATCCATGCCGTT |
| GAPDH | Forward | AGAACATCATCCCTGCATCCAC |
|  | Reverse | TCAGATCCACGACGGACACA |

**Raw Data of WB bands:**

^
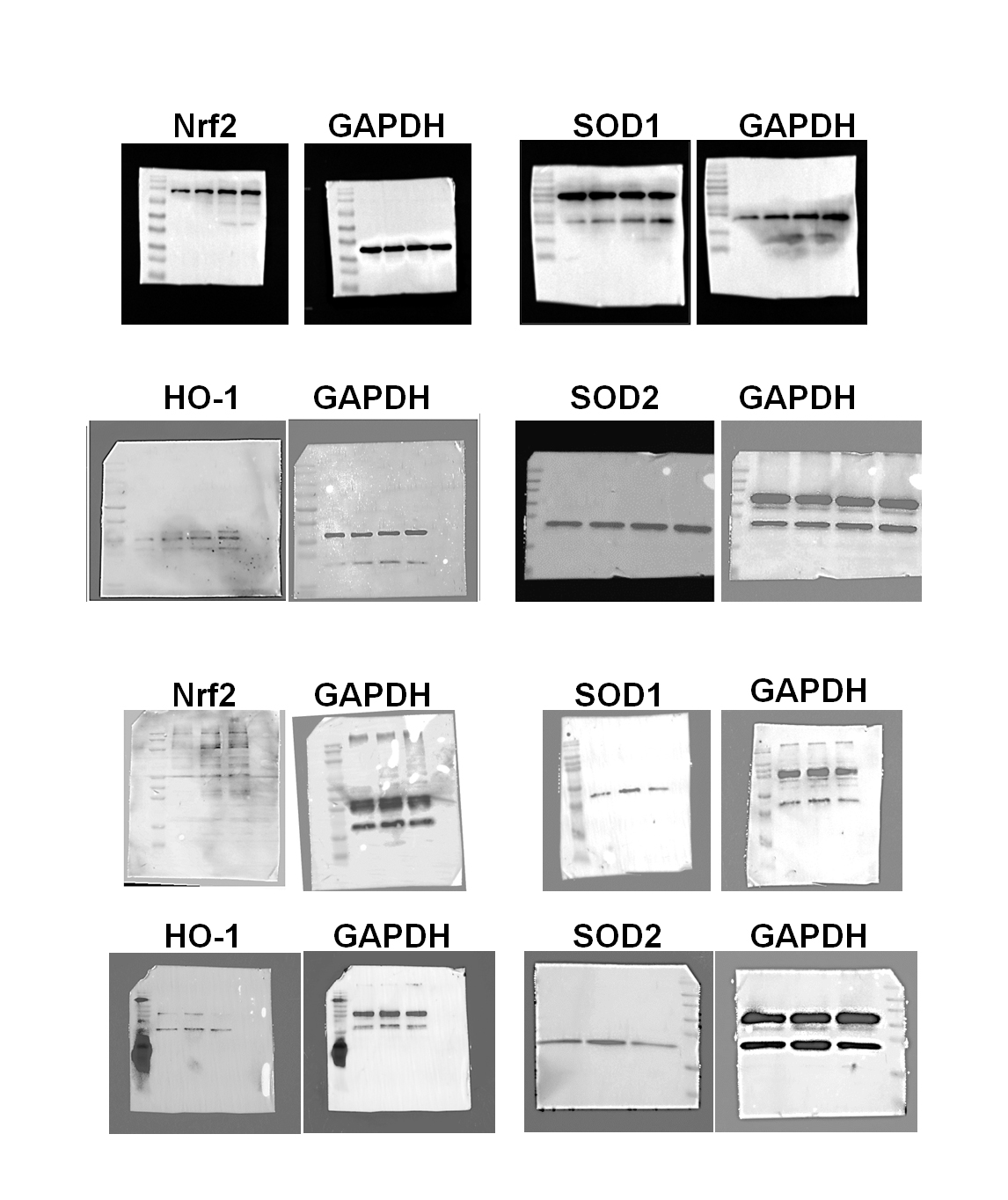
^

^
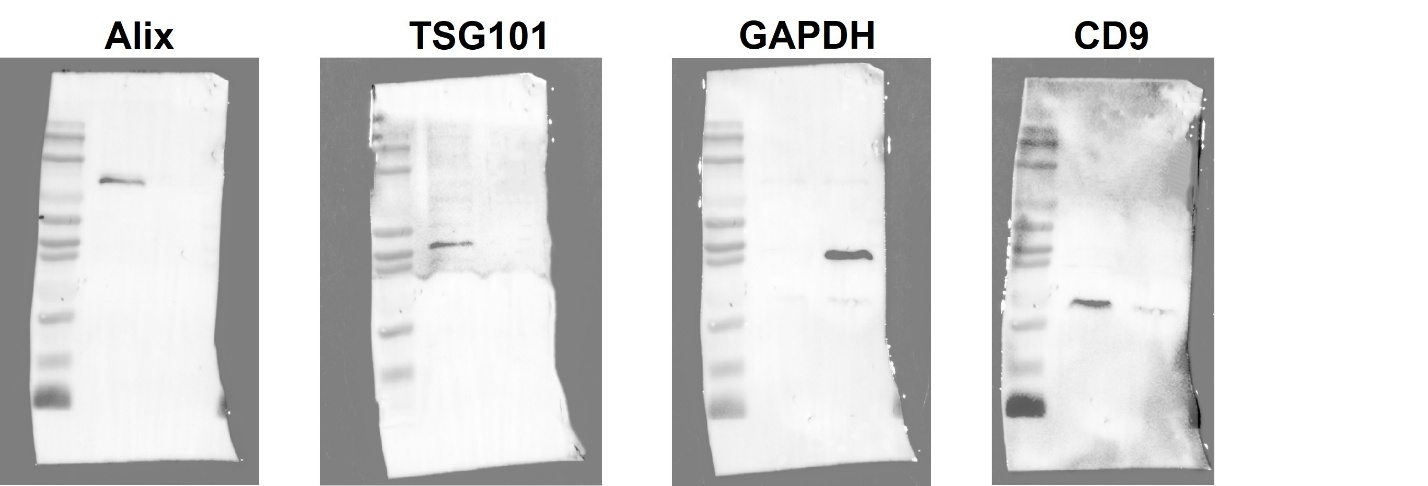
^
